# The transverse occipital sulcus and intraparietal sulcus show neural selectivity to object-scene size relationships

**DOI:** 10.1038/s42003-021-02294-9

**Published:** 2021-06-22

**Authors:** Lauren E. Welbourne, Aditya Jonnalagadda, Barry Giesbrecht, Miguel P. Eckstein

**Affiliations:** 1 Department of Psychological and Brain Sciences, University of California, Santa Barbara, USA; 2 Institute for Collaborative Biotechnologies, University of California, Santa Barbara, USA; 3 York NeuroImaging Centre, Department of Psychology, University of York, York, UK; 4 Electrical and Computer Engineering, University of California, Santa Barbara, USA; 5 Interdepartmental Graduate Program in Dynamical Neuroscience, University of California, Santa Barbara, USA

**Keywords:** Object vision, Perception

## Abstract

To optimize visual search, humans attend to objects with the expected size of the sought target relative to its surrounding scene (object-scene scale consistency). We investigate how the human brain responds to variations in object-scene scale consistency. We use functional magnetic resonance imaging and a voxel-wise feature encoding model to estimate tuning to different object/scene properties. We find that regions involved in scene processing (transverse occipital sulcus) and spatial attention (intraparietal sulcus) have the strongest responsiveness and selectivity to object-scene scale consistency: reduced activity to mis-scaled objects (either unusually smaller or larger). The findings show how and where the brain incorporates object-scene size relationships in the processing of scenes. The response properties of these brain areas might explain why during visual search humans often miss objects that are salient but at atypical sizes relative to the surrounding scene.

## Abstract (web summary)

Lauren Welbourne et al. use functional magnetic resonance imaging to investigate the neural dynamics linked to how humans process object size in the environment. After showing participants a series of images with appropriately-sized or misscaled objects (such as a giant toothbrush on a bathroom sink), the authors observed that the temporal occipital sulcus and intraparietal sulcus were strongly responsive to normally-sized, but not misscaled, objects, suggesting that object representations in both brain regions incorporate the objects’ typical size relationships to the surrounding scene.

## Introduction

Vision at the point of fixation, the foveola, is processed with high-spatial resolution^1^ and is not degraded by patterns flanking a target (e.g., crowding^2^). Vision away from the point of gaze, in the visual periphery, is processed with less spatial detail and is subject to crowding^3^. A foveated visual system reduces the brain’s computational cost but must rely on several strategies to ensure successful search for objects in scenes. Guided eye movements to point the high-resolution fovea to regions in a scene are critical to acquiring task-relevant information^4–6^. Humans rely on statistical relationships between the object searched and the scene to guide eye movements and make search decisions^7–9^. For example, the brain rapidly processes information about a scene, including its category (indoor, outdoor, natural, city, etc.^10^), the configuration of objects^11–15^, and highly visible objects that often co-occur with the target^16^. This information is then used to direct eye movements towards locations expected to contain the searched object^10,11,14,17–19^. In addition, humans also use scene information to guide attention towards target spatial sizes that are consistent with the scene^20^. For example, if one is searching for a pencil, then a scene with a desk, a computer, and a keyboard all provide information about the likely relative size of the pencil to the rest of the scene. When a target appears at an anomalous size, humans can often miss such targets, even if they are large and salient^20,21^. The tendency to miss inappropriately scaled objects is not a maladaptive human behavior but rather a by-product of a useful brain strategy to rapidly discount potential distractors that might look like the target but are not at the correct spatial scale.

The locus of cortical representations of statistical relationships in scenes, crucial for visual search, has been the focus of many studies. The parahippocampal place area (PPA) encodes important components of scenes, specifically layout and general geographical features of space^22,23^. Parahippocampal sub-regions^24,25^ also represent semantic and spatial associations between co-occurring objects (but see^26^). The expected location of a target object within a scene is represented in the lateral occipital complex (LOC), the intraparietal sulcus (IPS), and the frontal eye fields (FEF)^27^. In relation to object size, previous studies have shown that the physical size of real-world objects is represented in the occipitotemporal regions of the cortex, with an organization of real-world size preference across the ventral surface. Small objects are more strongly represented in the object region LO, whereas large objects are more strongly represented in scene regions (e.g. PPA)^28,29^.

To our knowledge, little is known about which brain areas are responsive to the spatial scale of an object relative to the surrounding objects in the scene. Such brain areas might play an important role in guiding attention during search toward likely target sizes^20^. We used functional magnetic resonance imaging (fMRI) to understand the responsiveness and selectivity of various brain regions to size relationships between an object and the surrounding scene while controlling for other properties of our stimuli, which also affect neural responses (BOLD activity). We will refer here to these size relationships as object-scene scale consistency. Subjects maintained gaze on a central fixation point while viewing computer-generated scenes that preceded the appearance of foveal objects onto the scene, which varied in size relative to the surrounding scene. To ensure attention was maintained, the subjects’ task was to detect blank trials in which no object appeared in the scene. We investigated activity in functionally-defined scene regions PPA, TOS, and retrosplenial cortex, RSC; object region LO; IPS, which is involved in spatial attention and eye movements; the fusiform face region FFA, as a control comparison; and an anatomically-defined early visual area, V1. We utilized general linear models (GLM) with a finite basis response function^30^ and multi-voxel pattern analysis (MVPA)^27,31^ to quantify the responsiveness of voxels and brain areas to object-scene scale consistency. To isolate the effects of object-scene scale consistency on BOLD activity from influences of real-world object (physical) size, object retinal image size, and scene field-of-view, we used a voxel-wise encoding model^32,33^. This method provided voxel responsiveness to the various object properties within the different brain regions of interest (ROIs), allowing us to identify the contribution of object-scene scale consistency to the BOLD time series while other properties were also taken into account. A comparison across feature weight values allowed separating overall responsiveness of an area to visual information from how selective a voxel was to a single feature rather than broadly tuned to multiple features^34–36^.

We found that scene region TOS and attention-related area IPS showed strong evidence of responsiveness and selectivity to object-scene scale consistency. Specifically, these regions responded more strongly to objects that were correctly scaled within a scene, than to objects that were mis-scaled relative to the scene, and they also contained voxels that responded more strongly to this feature (object-scene scale consistency) than to other properties (i.e. real-world object size, retinal size, or scene field-of-view). TOS and IPS may therefore play a role in the behavioral effects previously observed, when the target object is too large in size relative to the surrounding objects in the scene^20^.

## Results

### Regional brain responses are modulated by object-scene scale consistency

**Fig. 1:**
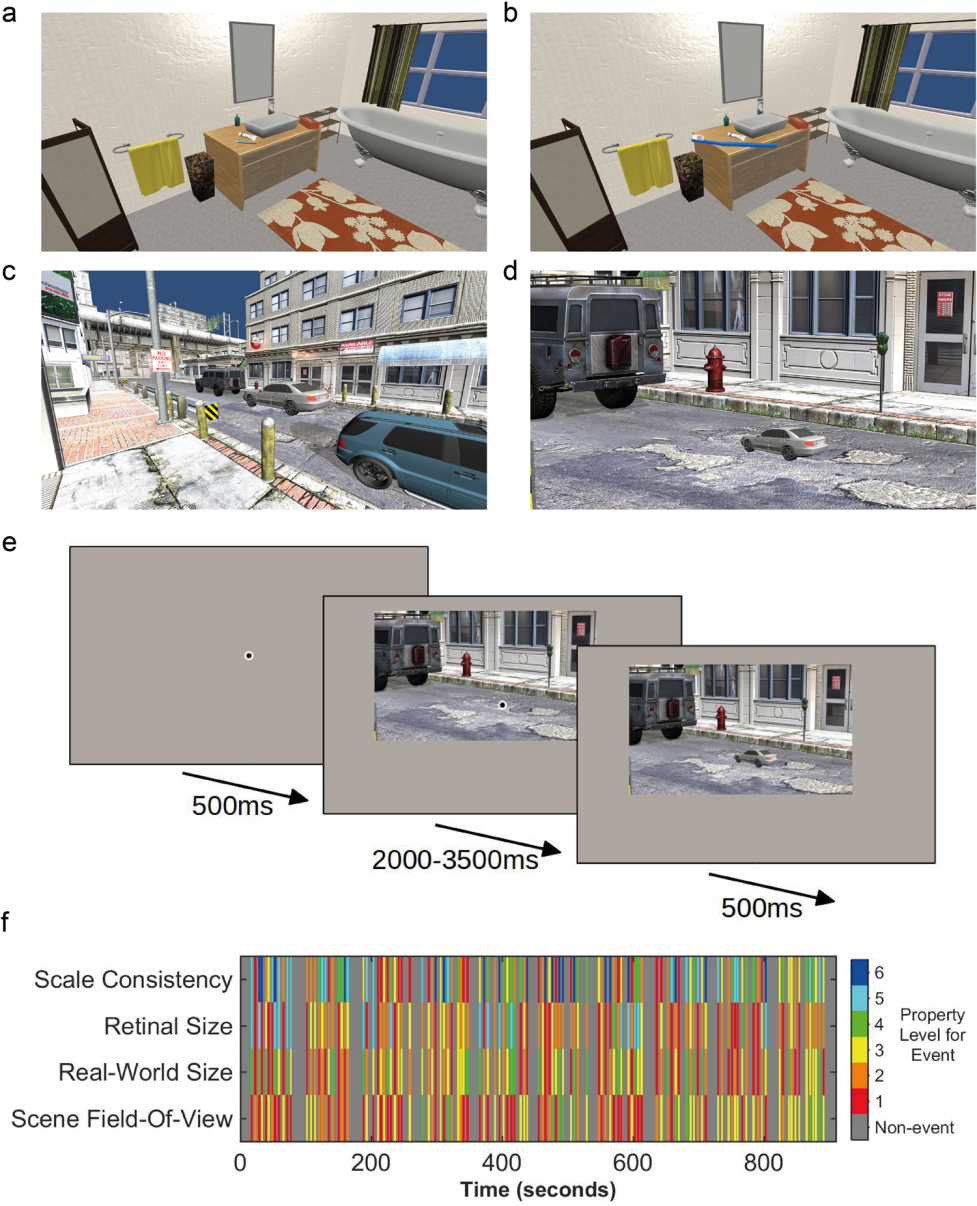
Stimulus examples and fMRI events. **a** Small object (toothbrush), normal object-scene scale consistency level. **b** Small object (toothbrush), maximum mis-scaled level. **c** Large object (car), normal object-scene scale consistency level. **d** Large object (car), maximum mis-scaled level. Images **a** and **b** show a retinal size manipulation (fixed scene field-of-view (FOV), with changing object retinal size). Images **c** and **d** show a scene FOV manipulation (fixed object retinal size, with changing FOV). **e** Schematic of a single trial presentation in the fMRI experiment. For visibility here, the central fixation point is shown larger than the actual size used and with a lighter gray outer ring (in the actual experiment the gray ring matched the background gray of the screen). **f** Example of the event order in a single scan. Each vertical multi-colored line indicates the onset time of an event, with the property levels for each of four properties that can be defined in the images (scale consistency, object retinal size, real-world size, scene field-of-view) indicated by color (see legend).

**Fig. 2:**
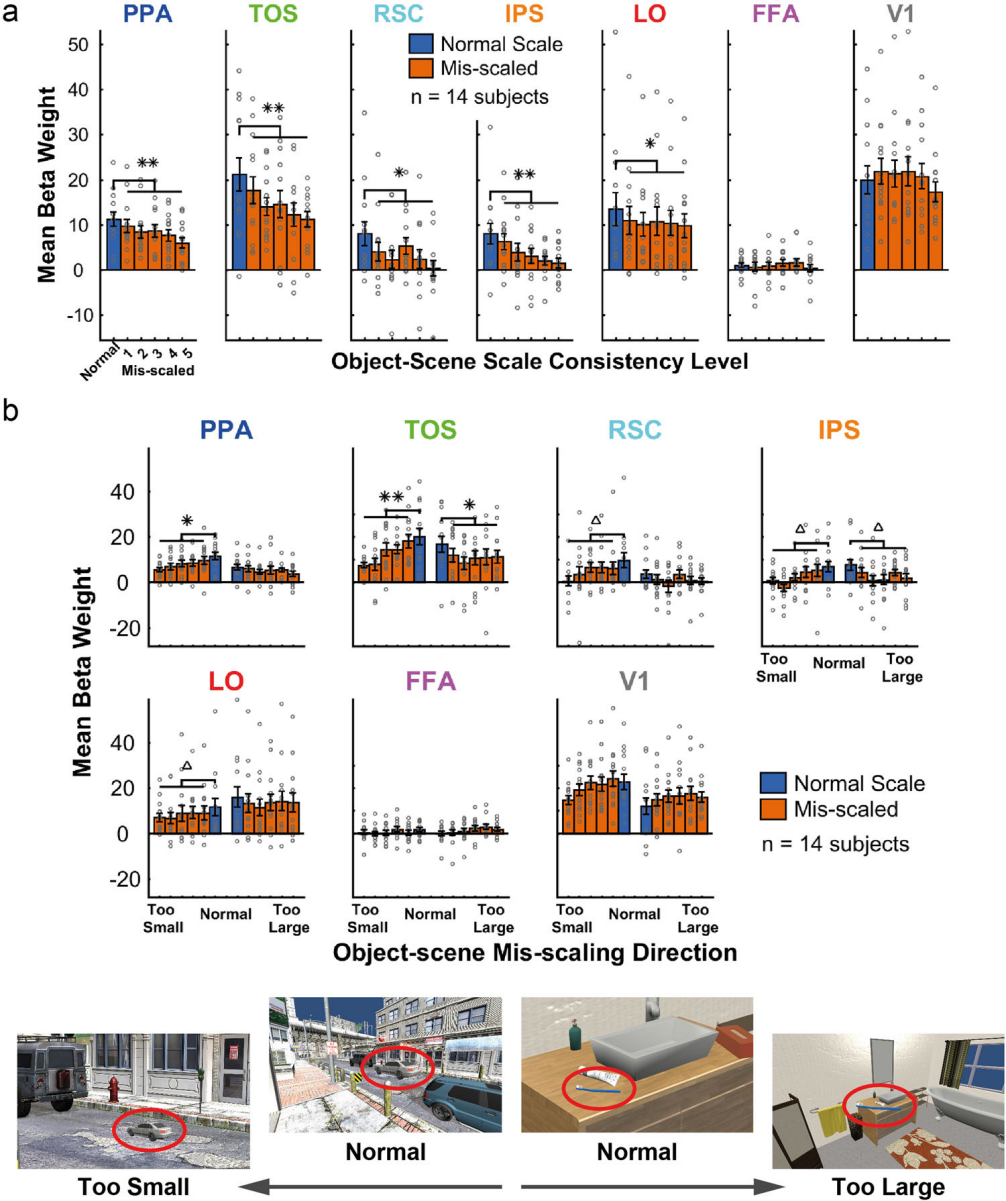
GLM results. Mean beta weights with SEM error bars (**a**) across object-scene scale consistency levels, in each ROI; **b** across object-scene scale consistency levels, where conditions are split by mis-scaling direction (half of the objects are in each mis-scaling direction). Significant differences between normal scale consistency levels and the mean of the mis-scaled levels are indicated above the relevant plots, tested for significance with permutation tests: **p < .001, *p < .01, ^Δ^*p* < .05 (1000 permutations, one-tailed, FDR corrected). Example images, with targets (circled red) corresponding to normal, too small, and too large, are shown below the plots. Error bars show the standard error of the mean (SEM). Individual subject data points are overlaid on the bars as gray circles.

We first used a univariate analysis (General Linear Model, GLM) to investigate the extent to which object-scene scale consistency evoked differential brain responses in the defined regions of interest (see Fig. 1 for stimulus examples and experimental design). Specifically, we assessed whether the normal scale condition produced a greater response than the mis-scaled conditions. In this first analysis (Fig. 2a), all ROIs, except FFA (*p* = .561) and V1 (*p* = .741), showed a reduction in BOLD responses for mis-scaled levels, with a higher GLM beta weight for the normal scale consistency level (*p* = .001 for LO and RSC, and *p* < .001 for TOS, PPA, and IPS, all p-values from permutation tests using 1000 permutations, one-tailed, with false-discovery rate (FDR) correction across ROIs). In a second univariate analysis, the data were split by the direction of mis-scaling (half of the 10 stimulus objects were mis-scaled to be too small, while the other half were mis-scaled to be too large). The purpose of this analysis was to determine whether each direction of mis-scaling produced the same effect (i.e., when the objects were either too large or too small). The results, shown in Fig. 2b, revealed that only TOS and IPS demonstrated this same pattern for both directions of mis-scaling (TOS, mis-scaled too small *p* < .001, mis-scaled too large *p* = .005; IPS, mis-scaled too small *p* = .036, mis-scaled too large *p* = .035 (1000 permutations, one-tailed, FDR corrected)). Whereas, PPA, RSC, and LO only reached statistical significance when large objects were mis-scaled to be smaller than normal (larger beta weights for the normal scale level) (*p* = .005, *p* = .036, and *p* = .036, respectively, 1000 permutations, one-tailed, FDR corrected). The mis-scaled levels were not significantly lower than the normal level for either mis-scaling direction in FFA and V1, or in one direction for PPA, RSC, and LO (FFA mis-scaled too large *p* = .128; FFA mis-scaled too small *p* = .189; V1 mis-scaled too large *p* = .999; V1 mis-scaled too small *p* = .070; PPA mis-scaled too large *p* = .109; RSC mis-scaled too large *p* = .128; LO mis-scaled too large *p* = .098; 1000 permutations, one-tailed, with FDR correction across all tests and ROIs). The data in Fig. 2 is available in Supplementary Data 1.

### Patterns of regional brain responses discriminate between levels of object-scene scale consistency

**Fig. 3:**
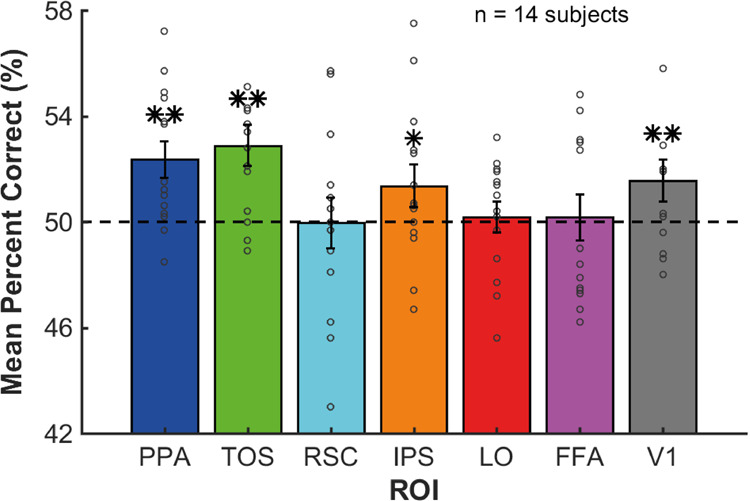
MVPA output. Mean percent correct (PC) (%) across subjects from the MVPA for each ROI. Permutation tests were performed to determine whether the mean PC values were significantly different from chance (50%). These were FDR corrected across ROIs: **p < .001, *p < .01, 1000 permutations, one-tailed, FDR corrected across ROIs. The dashed black line at 50% indicates chance level of percent correct responses. Error bars show the SEM. Individual subject data points are overlaid on the bars as gray circles.

To assess whether information about object-scene scale consistency was available, not only in the overall amplitude of the BOLD response but also in the activity patterns in the functionally-defined regions of interest, we conducted a multi-voxel pattern analysis (MVPA). The analysis was conducted on each subject in each ROI, and the goal was to predict the scale consistency of the object on each trial: normal scale vs. mis-scaled (see Methods). To reduce the feature dimensionality, the voxels used for each ROI in this analysis were those defined as the top activation voxels based on the pre-event scene presentation (across all trials) relative to the blank period baseline of each ROI (see Methods). The MVPA results (Fig. 3) were consistent with the univariate analysis, such that patterns of activity in areas TOS (*p* < .001) and IPS (*p* = .002) discriminated trials containing object-scene scale *consistent* objects from those that contained object-scene scale *inconsistent* objects. In addition, significant discrimination was also found in areas PPA (*p* < .001) and V1 (*p* < .001) (significance determined using permutation tests: 1000 permutations, one-tailed, FDR corrected across ROIs). Areas RSC, LO, and FFA did not reach statistical significance (*p* = .490, *p* = .396, and *p* = .396, respectively; 1000 permutations, one-tailed, FDR corrected across ROIs). The data in Fig. 3 is available in Supplementary Data 2.

### Voxel-wise encoding models reveal feature selectivity to object-scene properties

**Fig. 4:**
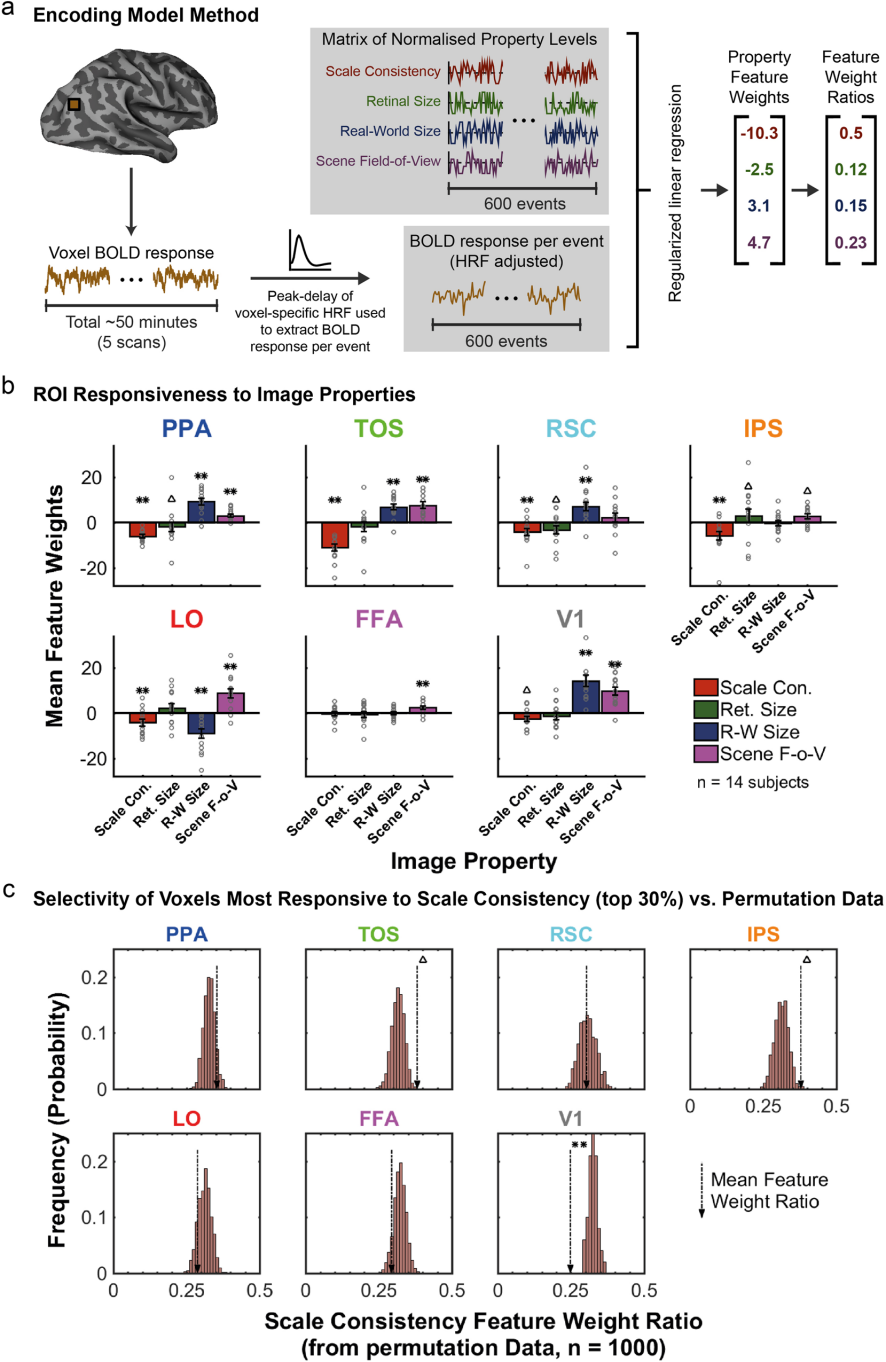
Encoding model method and output. **a** Schematic of the encoding model method used to produce property feature weights. For each voxel, the linear-detrended BOLD responses for each event in each of the five scans were extracted using the peak-delay of the voxel-specific HRF estimate (see Methods). Property levels for each scan were normalized between −1 and 1, and then entered into a regularized linear regression with the event BOLD responses from the voxel to produce feature weights for each property, and subsequently, the ratio of absolute feature weights was calculated. **b** Mean feature weights across voxels and subjects for each property in each ROI, with SEM error bars. Individual subject data points are overlaid on the bars as gray circles. Values were tested for significance against zero using permutation tests: **p < .001, ^Δ^*p* < .05, 1000 permutations, two-tailed, FDR corrected across properties and ROIs. **c** Histograms of the permutation data for the Scale Consistency feature weight ratio, when using the top 30% of voxels most responsive to scale consistency. The mean feature weight ratio for scale consistency from the actual data is indicated by dashed arrows, with significance indicated where relevant: **p < .001, ^Δ^*p* < .05, 1000 permutations, two-tailed, FDR corrected across ROIs.

Our univariate and multivariate analyses evaluated the responsiveness to object-scene scale consistency within different functionally-, and anatomically- (V1), defined areas. Yet, those analyses do not explicitly account for other properties of the stimuli that also varied as we manipulated object-scene scale consistency (e.g., retinal size, scene field-of-view). To isolate responsiveness and selectivity to object-scene scale consistency from other object/image properties that co-varied with the manipulation, we used a voxel-wise encoding model. Such models attempt to account for the time course of BOLD responses in a voxel in terms of a combination of a set of feature values, which can be the response levels of a number of hypothetical receptive fields^33^ or image features/categories^32^. The resulting weights for each feature that maximize the model’s prediction of the BOLD response for the voxel are indicative of the responsiveness of the voxel to the feature. A voxel-wise encoding model was therefore applied to estimate the contribution of each of the object properties (the features) to the fMRI signal within each ROI voxel for each subject (see Fig. 4a for a schematic illustration of the encoding model method). The analysis utilized four object properties: scale consistency, retinal size of the object, real-world size, and scene field-of-view (see Methods).

To determine the overall responsiveness of an area to each of the features, we first tested whether the mean voxel feature weights were significantly different from zero using permutation tests (see Methods for details). Figure 4b shows the mean feature weights for each property across all voxels in each ROI. The results are consistent with those obtained with the univariate GLM and MVPA methods but with higher statistical power. All areas that showed significant responsiveness to scale consistency in any of the GLM and MVPA methods (TOS, PPA, IPS, RSC, LO, V1), resulted in a significant mean feature weight for scale consistency in the encoding model (all *p* < .001 except V1 where *p* = .015, 1000 permutations, two-tailed, FDR corrected across all properties and ROIs). The negative value for the scale-consistency feature weights signifies that the BOLD activity is reduced with increasing scale inconsistency between the target object and surrounding objects/background. Furthermore, the results show that the different areas are also significantly responsive to other feature properties: object retinal size, real-world object size, and field-of-view. For example, TOS also resulted in statistically significant positive feature weights for increasing real-world size and scene field-of-view (both *p* < .001, 1000 permutations, two-tailed, FDR corrected across all properties and ROIs), i.e., a greater response to larger real-world object sizes and scene FOVs. LO resulted in a positive feature weight for scene field-of-view but negative feature weight for real-world size (both *p* < .001, 1000 permutations, two-tailed, FDR corrected across all properties and ROIs), i.e., a greater response to larger scene FOVs and to smaller object sizes (a decreased response to larger real-world object sizes). Other properties reaching significance for the ROIs are as follows: PPA, scene FOV *p* < .001; PPA, retinal size *p* = .014; PPA real-world size *p* < .001; RSC, retinal size *p* = .025; RSC, real-world size *p* < .001; IPS, scene FOV *p* = .025; IPS, retinal size *p* = .042; LO, scene FOV *p* < .001; LO, real-world size *p* < .001; FFA, scene FOV *p* < .001; V1, scene FOV *p* < .001; V1, real-world size *p* < .001 (each using 1000 permutations, two-tailed, FDR corrected across all properties and ROIs).

Some properties did not reach statistical significance, as follows: TOS, retinal size *p* = .226; RSC, scene FOV *p* = .102; IPS, real-world size *p* = .788; LO, retinal size *p* = .102; FFA, scale consistency *p* = .719; FFA, retinal size *p* = .506; FFA, real-world size *p* = .850; V1, retinal size *p* = .080 (each using 1000 permutations, two-tailed, FDR corrected across all properties and ROIs). Weight magnitudes depend on the voxel’s responsiveness to the feature but also on the overall responsiveness of a brain area to the visual stimuli. For example, V1 responds highest to visual information, resulting in a significant feature weight for object-scene scale consistency but rather small compared to other feature weights. To measure whether voxels are selective to a single feature or broadly responsive to all the features^34–36^, irrespective of the area’s overall response to the visual stimuli, we estimated responsiveness to object-scene scale consistency *relative* to other features. We concentrated on voxels with the highest responses to the object-scene scale consistency rather than include in our analysis voxels that were not very responsive. Are voxels that are highly responsive to changes in scale consistency less responsive to other features (high selectivity) or are they broadly tuned to many properties (low selectivity)?

Using absolute values, we computed the ratio of the scale consistency feature weight to the sum of all feature weights for just those voxels that were highly responsive to scale consistency (for each area the top 30% most responsive voxels to scale consistency, see Methods). If we were using all voxels, an average feature weight ratio of 0.25 would be expected by chance. However, because we are subsampling the voxels with high responsiveness to object-scene scale consistency, we expect statistically that the average feature weight ratios be larger than 0.25 just by chance. Therefore, we used the permutations and extract feature weight ratios in exactly the same way as we did for the actual data (i.e., for the top 30% of voxels most responsive to scale consistency, from every permutation, see Methods) and assess the statistical significance of voxel selectivity. Figure 4c shows histograms from the permutation data of the average voxel feature weight ratio for scale consistency expected by chance. The dashed arrow for each ROI indicates the mean feature weight ratio for scale consistency (in the top 30% of voxels most responsive to scale consistency) observed in the actual data. ROIs TOS and IPS resulted in a statistically significant indication of selectivity to object-scene scale consistency (TOS *p* = .014, IPS *p* = .028, 1000 permutations, two-tailed, FDR corrected across ROIs). This indicates that the voxels most responsive to scale consistency within these two ROIs were also particularly narrowly tuned to scale consistency over the other properties (retinal size, real-world size, and scene field-of-view). In V1, the mean feature weight ratio for scale consistency was significantly lower than expected by chance (*p* < .001, 1000 permutations, two-tailed, FDR corrected across ROIs). This indicates that the voxels most responsive to scale consistency in V1 were still significantly *more* responsive to one or more of the other properties than to scale consistency (in line with the larger feature weights produced for real-world size and scene FOV across all voxels in V1, shown in Fig. 4b). The selectivity to scale consistency in all of the other ROIs did not reach statistical significance (PPA, *p* = .129; RSC, *p* = .467; LO, *p* = .163; FFA, *p* = .129; 1000 permutations, two-tailed, FDR corrected across ROIs). The data in Fig. 4 is available in Supplementary Data 3.

**Fig. 5:**
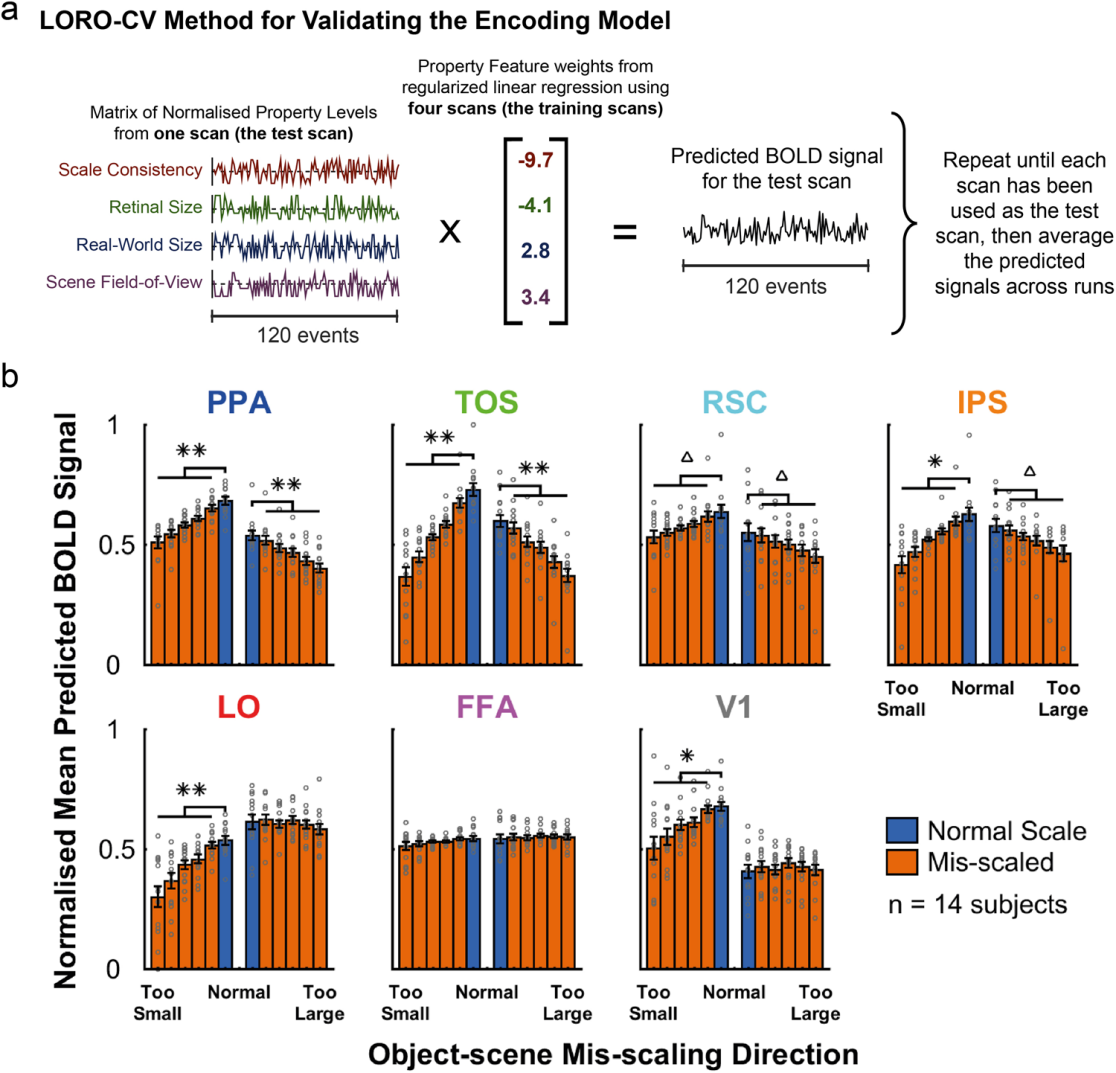
Predicted BOLD signals using Encoding Model Feature Weights. **a** Schematic of the LORO-CV method used to validate the encoding model. **b** Mean predicted BOLD signals for each ROI—normalized across subjects and ROIs—for each scale consistency level, when split by object scale direction (plotted in the same manner as the GLM data in Fig. 2b). Significance tests (paired t-tests, one-tailed) were performed between the normal scale levels and the mean of the corresponding mis-scaled levels, FDR corrected for multiple comparisons across all tests and ROIs: **p < .001, *p < .01, ^Δ^*p* < .05. Error bars show the SEM. Individual subject data points are overlaid on the bars as gray circles.

To assess the validity of the encoding model, we evaluated whether we could predict the average voxel BOLD response per ROI for each scale consistency condition (analogous to the univariate GLM analysis, in Fig. 2b) from the encoding model’s feature weights and the feature values of each image presented during the fMRI runs. Presumably, the univariate GLM analysis on voxel response for each area (Fig. 2b) results from the contributions of the various features to the BOLD activity. Thus, the encoding model should be able to predict the variations on mean BOLD activity across scale-consistency conditions for each brain area. We used a Leave-One-Run-Out Cross-Validation (LORO-CV) method to generate predictions for the BOLD signals in each voxel for each scan run (see Methods, and schematic in Fig. 5a). The normalized data are plotted in Fig. 5b, using the same plotting style as in Fig. 2b. We used paired t-tests to determine whether the predicted BOLD signal for the normal scale level was significantly greater than the mean of the mis-scaled levels, in each mis-scaling direction for each ROI. We observed a significant difference in both scaling directions for TOS (mis-scaled too small: t(13) = 6.722, *p* < .001; mis-scaled too large: t(13) = 6.643, *p* < .001), PPA (mis-scaled too small: t(13) = 7.198, *p* < .001; mis-scaled too large: t(13) = 6.539, *p* < .001), IPS (mis-scaled too small: t(13) = 3.581, *p* = .003; mis-scaled too large: t(13)=2.159, *p* = .035), and RSC (mis-scaled too small: t(13) = 2.428, *p* = .027; mis-scaled too large: t(13) = 2.154, *p* = .035), and for just one direction (large objects that were mis-scaled to be too small) in LO (t(13) = 5.162, *p* < .001), and V1 (t(13) = 3.939, *p* = .002) (all tests one-tailed with FDR correction across all tests and ROIs). All remaining ROIs/directions did not show a significant difference between normal and mis-scaled levels, as follows: FFA, mis-scaled too small: t(13) = 1.273, *p* = .143, mis-scaled too large: t(13) = −0.543, *p* = .321; LO, mis-scaled too large: t(13)=0.269, *p* = .396; V1, mis-scaled too large: t(13) = −0.851, *p* = .239 (one-tailed paired t-tests, FDR corrected across all tests and ROIs). The data in Fig. 5 is available in Supplementary Data 4.

The predicted BOLD data from the encoding model show similar trends to the empirical BOLD responses obtained from the human fMRI data (Fig. 2b). The results for TOS, IPS, LO, and FFA were consistent across the predictions for the encoding model and those empirically measured.

For other areas the trends agreed, yet, there were some differences. The encoding model predicted that both directions should be significant in PPA and RSC (both of these ROIs showed significance in only one scaling direction in the human data). It also predicted a significant reduction in BOLD signal for large objects mis-scaled to be too small in V1, which did not reach significance in the human data.

## Discussion

To efficiently search, humans rely on statistical relationships between objects and scenes. The human brain rapidly processes the scene type, objects in the scenes, and their configuration and utilizes such information to guide eye movements and search decisions^7–10,12,14,19,37,38^. Furthermore, a recent study has shown how scene information is also used to guide attention to likely sizes of the searched object^20^. When the searched object unexpectedly appears at an inconsistent scale relative to the scene, then observers often miss the targets. Little is known about the brain areas that might mediate such an object-scene scale consistency influence on search. Here, we used fMRI to assess which brain areas are responsive—and selective—to object-scene scale consistency, utilizing various analysis techniques: GLM, MVPA, and a voxel-wise encoding model.

In order to best understand the underlying influence of object-scene scale consistency within each functional (and anatomical) brain region, the findings from each analysis must be interpreted together. Table 1 shows a summary of all the results from the current study. Across the GLM, MVPA, and voxel-wise encoding model, regions TOS and IPS show the most consistent indication of responsiveness and neural selectivity (high responsiveness relative to the other features) to object-scene scale consistency: (1) brain responses are significantly lower when objects are *inconsistent* in spatial scale relative to the surrounding scene (in the GLM analysis), (2) significant prediction accuracy for distinguishing normally-scaled vs. mis-scaled objects (MVPA), and (3) significant responsiveness *and* selectivity to the scale consistency property were observed after accounting for the contribution of other object properties (voxel-wise encoding model). The control region, face region FFA, did not show any indication of responsiveness to object-scene scale consistency in any of the three analyses (Table 1).

**Table 1:** Summary of results from each analysis.

Brain Area	GLM	GLM: Direction of Mis-scaling		MVPA	Encoding Model Responsiveness and Selectivity to Scale Consistency	
		Too Small	Too Large		Responsiveness: Feature Weight	Selectivity: Feature Weight Ratio
TOS	**✓**	**✓**	**✓**	**✓**	**✓**	**✓**
IPS	**✓**	**✓**	**✓**	**✓**	**✓**	**✓**
PPA	**✓**	**✓**	**✗**	**✓**	**✓**	**✗**
LO	**✓**	**✓**	**✗**	**✗**	**✓**	**✗**
RSC	**✓**	**✓**	**✗**	**✗**	**✓**	**✗**
FFA	**✗**	**✗**	**✗**	**✗**	**✗**	**✗**
V1	**✗**	**✗**	**✗**	**✓**	**✓**	**✗**

Ticks indicate that the relevant statistical test was significant to at least *p* < .05 (with FDR correction), whereas crosses indicate a non-significant result. See Results section for details of each test.

Other ROIs (PPA, RSC, LO, and V1) also showed some responsiveness to scale-consistency but were more inconsistent (Table 1). Region PPA was modulated by scale consistency in the MVPA, the GLM analysis when the object became smaller relative to the scene, and the responsiveness measure of the feature encoding model. Areas LO and RSC showed a statistically significant modulation in the GLM for objects mis-scaled to have a smaller relative size to the scene and in the responsiveness measure of the feature encoding model, but not for the MVPA analysis nor the GLM analysis for objects becoming inconsistently large relative to the surrounding scene. Area V1 showed no statistically significant modulation in any of the GLM analyses but did show significant modulation in the MVPA, as well as in the responsiveness measure of the feature encoding model. The feature encoding model showed no statistically significant selectivity for object-scene scale consistency relative to other features for any of these four areas: PPA, LO, RSC, and V1.

Are there other plausible explanations for the reported findings of neural responsiveness to object-scene scale consistency? For example, could the areas be merely responding to an odd-ball or surprise rather than scale consistency? There are several indications that this is not the case. First, the vast majority of cognitive neuroscience studies measuring responses to an odd-ball results in an increase in an evoked potential^39^ or BOLD activity^40,41^, while we find a reduction in activity to the mis-scaled objects. Second, odd-ball paradigms typically use a low prevalence for the odd-item^42–44^ (e.g., 20 %) while our mis-scaled objects were presented more frequently. Third, we do not find the same effect in all our brain areas, indicating that this is not just a general response to odd-ball/surprise stimuli, which might be expected to be present across a more extensive set of brain regions.

Instead, we suggest that the effects are mediated by excitatory interactions across objects. This has been suggested for objects that are semantically related or that spatially co-occur^25,45^ and mediate increases in neural decoding accuracy of searched objects^46,47^ and behavioral search performance^7,8,13,17,48^. The current work suggests a similar mechanism for objects that share relative sizes consistent with their frequent occurrence in the visual world.

Such mechanisms would be consistent with the computational framework of optimal Bayesian models in which probabilities of the detection of other objects multiplicatively excite or inhibit the estimated probabilities of a target or object being present. Such a mechanism has been applied to model covert attention^49,50^ and also search for objects in scenes^10,17^, as well as shown to improve computer vision models^51^. In this framework, the neural responses tuned to an object are modulated in an excitatory manner by the responses to surrounding objects that appear at relative sizes that frequently occur in the natural world.

Alternatively, the enhancement could be explained in terms of a proposed theory in which individual objects compete for neural processing of resources^45,52^ in a similar manner as the biased competition of attention^53^. Adaptations to the typical location of objects and their relative size would contribute towards reducing inter-object competition and by integrating multiple objects into group representations^45,52^.

The feature encoding model also estimated weights for the three other features. TOS, PPA, RSC, and V1 all showed statistically significant positive feature weights for increasing object real-world size. This is consistent with previous results showing scene regions TOS and PPA responding preferentially to objects with a large real-world size, including when small objects are perceived to be large^29,54–56^. Perhaps, most surprising is the responsiveness of V1 to the real-world size of objects. Other studies have decoded other high-level properties such a natural scene categories from V1/V2 scenes^57^. One possible explanation is some mid-level property differences that co-varies with real-world object size^58^. Another explanation is feedback from high-level areas such as those mediating perceived size^59^ and categorization of natural scenes^57^.

For area LO, the encoding model resulted in a significant negative feature weight for real-world object size, consistent with previous studies showing that LO typically responds strongly to all conceptual size representations of small objects^28,60^ and large real-world objects when they were perceived to be small, due to perceived distance or the proximity to a subject’s hand^54,55,61^.

Almost all areas were responsive to the scene field-of-view. The increase of activity with the field-of-view in scene-selective areas is consistent with previous studies that have shown separate modulation with increasing space and clutter of the scenes^62^. In addition, increasing the field-of-view will increase the perceived distance and change the object’s relative size to the surrounding objects. Both of these properties will increase the object’s perceived size, which has been shown to modulate fMRI activity. Studies have shown that V1 responds to the perceived size of objects, e.g., in size illusions^63–65^, and receives feedback from extrastriate regions^48^. This would predict larger V1 activity with increasing scene field-of-view, which is consistent with our results of positive V1 feature weight from the encoding model. PPA and TOS activity increase with larger perceived size/distance^54^ and is also consistent with the obtained positive feature weights from the encoding model. Thus, some of the effects of scene field-of-view might be mediated by influences of perceived size.

However, there are some inconsistencies with past results. We obtained LO activity increasing with scene field-of-view, while Park et al.^62^ showed a reduction. The presence of a central object in our images and its absence in the Park et al.^62^ scenes might explain the discrepancy.

The encoding model also shows small but significant responsiveness to the object’s retinal size in some ROIs, with PPA and RSC responding to objects with small retinal sizes (as indicated by the negative feature weight). In contrast, no significant responsiveness to object retinal size was found for TOS. These results might seem to be inconsistent with previous studies showing that PPA and TOS, but not RSC, have increased response with object retinal size^56^. However, Troiani et al.^56^ only found an increased response with larger object retinal size when the objects were present with no backgrounds. For objects in scene backgrounds, more similar to our images, they found no effect of object retinal size^56^. Our study also found a significant positive feature weight for retinal size in IPS (responding to objects with larger retinal size), consistent with previous results showing IPS responsiveness to retinal size with simpler stimuli^66^.

For a number of areas with moderate responsiveness to object-scene scale consistency, we obtained varying results for the three analysis techniques. What accounts for such differences? The MVPA and GLM discrepancies might be explained by the fact that the two analyses are based on different voxels within an area. GLM averages across all the voxels in a functionally segmented brain area. In MVPA, a subset of most active voxels is sampled to reduce the dimensionality of the data and the number of estimated weights. For regions that carry discriminative information in all voxels in the area, voxel subsampling will likely have little effect, and GLM and MVPA techniques will tend to agree.

What might account for the differences between the GLM and Feature Encoding Model results? The manipulation of object-scene scale consistency required either changing the object’s retinal size while keeping the scene constant or changing the scene field-of-view while maintaining the retinal size of the object. Thus, the variations of BOLD response for a given brain region across the levels of object-consistency levels result from the combined effects of the various features on neural activity. The resulting feature weights from the encoding model explain some of the discrepancies between the results from the GLM analysis. In the GLM analysis, LO showed a significant modulation of activity when the object became inconsistently small relative to the scene, but not when it became inconsistently large. This result can be explained by the feature weights associated with LO (Fig. 4b). For size inconsistent objects that are too large, the expected reduction in LO’s BOLD activity from diminished scale consistency (negative feature weight for scale consistency) is offset by increased LO activity related to increasing FOV (larger positive feature weight for FOV). This results in a net null effect across scale-consistency levels (Fig. 2b).

The feature weights for real-world object size can also explain some of the GLM results. There are asymmetries in responsiveness to the normal-sized objects for areas PPA, RSC, V1, and LO (left vs. right blue columns in Fig. 2b). This can be explained in terms of the objects utilized for the scale manipulations, i.e., objects mis-scaled to be too large versus those mis-scaled to be too small. To create scale-inconsistent small objects (i.e., mis-scaled to be too small) that were visible after reducing the retinal size in the retinal-size manipulation, the objects that were mis-scaled to be too small were all objects with a large real-world size (50% of images). In contrast, to create scale-inconsistent large objects (i.e., mis-scaled to be too large) that did not become too large in retinal size when mis-scaled, we used objects with a small real-world size (50% of images). The interaction between the real-world object sizes used for the manipulation of the mis-scaling direction, and each area’s responsiveness to real-world object size, may contribute to the GLM results; for PPA, RSC, and V1 the positive feature weights for real-world size can explain the higher responsiveness for scale consistent objects that are large in real-world size (left vs. right blue columns in Fig. 2b). In contrast, LO’s negative feature weight for real-world object size can explain its higher responsiveness to small real-world object size (right vs. left blue columns in Fig. 2b).

To quantitatively formalize the process of predicting the GLM results in terms of the encoding model, for each brain region we generated normalized BOLD activity predictions across events using a Leave-One-Run-Out Cross-Validation (LORO-CV) method (Fig. 5b). Comparison of Fig. 5b and Fig. 2b suggest that the GLM results for scale-inconsistent small and large objects can be effectively explained for many of the brain areas and conditions in terms of the additive effects of the various features. There were three exceptions that showed similar trends but differed in the statistical results from the human results: the predictions for PPA and RSC activity for small objects mis-scaled to be inconsistently large, and V1 activity for large objects mis-scaled to be inconsistently small, which all reached statistical significance in the predictions, while they did not for the human observers (Fig. 2b).

To summarize, although we find responsiveness to object-scene scale consistency in a number of areas involved in object (LO) and scene (RSC, PPA) processing, scene region TOS and attention-related area IPS showed the strongest evidence of neural responsiveness and selectivity: they respond more strongly to an object when the object-scene scale is consistent, versus when the object is mis-scaled relative to the scene, and also contain voxels that respond more strongly object-scene scale consistency than the other properties. These regions may therefore play a role in the behavioral effects previously observed, whereby performance in visual search tasks is poorer when the target object is shown to be too large (mis-scaled)^20^. This finding expands on recent literature that explores the selectivity of these regions to object properties related to conceptual and physical size^28,29,54,55^, in addition to their traditional category specificity, i.e., responding preferentially to scenes and scene properties (e.g., layout) over other categories such as objects or faces^22,23^. The implementation of encoding models can reveal the brain areas’ joint tuning to multiple object and scene properties and also help explain the results of more traditional fMRI analysis (i.e., GLM).

## Methods

### Subjects

Fifteen subjects completed the fMRI tasks in this experiment. One subject was excluded from the analysis due to high levels of movement (between 1.5 and 6 mm of movement in every scan) and, critically, a lack of attention due to sleeping during the scanning sessions. All other subjects were included in the fMRI analysis (*n* = 14); the mean age was 22.8 years (range 19−28), and there were 8 female and 6 male subjects. A further 240 subjects were recruited via Amazon Mechanical Turk for an object-scene scale consistency rating task, used to acquire perceived scale consistency ratings to validate the levels in our object-scene scale consistency property. The Human Subjects Committee at the University of California, Santa Barbara approved these experiments, and all subjects provided informed consent to participate in the experiments.

### Stimulus images

The stimuli used in these experiments were computer-generated using Unity (Unity Technologies) and GIMP v2.8 (https://www.gimp.org/) software. Target objects were directly embedded within scenes, rather than using other cues of distance and object size (e.g., a Ponzo illusion). In order to control for the effects of object retinal size, the direction of size change during mis-scaling, and change in the field-of-view of the background scene, a number of properties were manipulated. A total of 10 different target objects were used: five increased in size when mis-scaled, and five decreased in size when mis-scaled. The practicalities of producing these images required smaller real-world objects to be used when the size needed to be increased (toothbrush, bedside lamp, teacup, computer mouse, and frying pan), and larger real-world objects were used when the size needed to be decreased (sofa, car, bus shelter, playground slide, and pool table). Each object was presented at six different scale consistency levels: 1 normal scale consistency level, and five mis-scaled levels, of increasing scale inconsistency relative to the scene; see details below on how these objective scale consistency levels were created. Furthermore, two different manipulations were used for each target object: (1) retinal size manipulation: the background scene image was fixed, and the change in object-scene scale consistency was created by adjusting the retinal size of the objects, and (2) scene field-of-view (FOV) manipulation: the retinal size of the objects was fixed, and the change in object-scene scale consistency was created by adjusting the field-of-view of the background scene. These conditions resulted in 12 different images for each target object and 120 different images in total – 20 for each of the six object-scene scale consistency levels.

The maximum mis-scaling of each object was x4 smaller/larger than the normal size of the object, and each of the six scale consistency levels was taken from a linear distribution between the normal and maximum mis-scaled size. In the retinal size manipulation, the scale of the object was increased/decreased by each linear step up to the maximum x4 mis-scaled level (original size manipulated by a factor of: 1, 1.6, 2.2, 2.8, 3.4, and 4); this was done within the GIMP software using isolated versions of the original size target objects exported from Unity. For the scene field-of-view manipulation, the object mis-scaling was achieved by adjusting the field-of-view (FOV) of the scene within the Unity software – the starting FOV size of the scene was increased or decreased by up to a factor of 4, using the same linear steps above, depending on whether the object was to appear to increase or decrease in size. In each case, the object and scene layers within the Unity file were saved in isolation and then re-combined within GIMP (following the necessary scale adjustments in the retinal size manipulation). Examples from each of the manipulations are shown in Fig. 1a–d. When viewed in the MRI scanner, the image size was set at 10° (width) by 5.84° (height) of visual angle. The stimuli were presented using MATLAB R2016b (The MathWorks Inc., Natick, MA, USA) and the Psychophysics Toolbox^67^.

### Object properties

In addition to the object-scene scale consistency property (scale consistency), three other object properties were assigned to each image. These were considered to be the primary high-level properties that differed between images and conditions, specifically: object retinal size, real-world object size, and scene field-of-view (FOV). For each property, the images were grouped into 4 or 5 levels, so that each image had a level assigned for every property. The images were designed to minimize correlations across the various other object properties with the object-scene scale consistency property. To determine whether the scale consistency levels were independent of the other object properties, we calculated Spearman rank correlations. Correlations between scale consistency and the other object properties were low and not statistically significant: with object retinal size (*r*_s_(118) = −0.0227, *p* = .8060), real-world object size (*r*_s_(118) = 0, *p* = 1), or scene field-of-view (*r*_s_(118) = −0.0840, *p* = .3616). All other correlations between the remaining object properties reached statistical significance (the full table of correlations can be seen in Supplementary Table 1). To avoid false-negative (Type II) errors, the significance values were not corrected for multiple comparisons; a conservative estimate of significance following correction for multiple comparisons may lead us to underestimate the relationship between scale consistency and the other properties (the significance of inter-correlations between the other properties is inconsequential to our analysis here). See Supplementary Table 1 for the output of the correlations, and Supplementary Figure 1 for scatter plots of the raw property values (prior to the grouping into levels) plotted as a function of the scale consistency level for every image.

Object retinal size for each image was calculated based on the known presentation size and viewing distance of the stimulus, using the diagonal size of the object (within a 2D bounding box rectangle). The scene FOV was taken from the properties of the 3D Unity scene. The real-world object size was estimated in inches based on the average of six representative real-world target objects (using the diagonal size through the 3D volume), which were found using search engines and product websites.

To confirm whether the scale consistency property levels were representative of a *perceived* change in scale consistency, independent ratings were acquired from an additional 240 subjects, using Amazon Mechanical Turk and Qualtrics (Qualtrics, Provo, Utah, USA). A Spearman rank correlation was performed between the grouped levels acquired from this experiment and the actual scale consistency property levels; perceived scale consistency was highly correlated with the actual scale consistency property (*r*_s_(118) = 0.8799, *p* < 10^−39^). For the rating task, the images were counterbalanced across six groups of 40 subjects, such that no target object was seen at more than one scale level by the same group of subjects; this resulted in 40 ratings per image. Subjects were provided with the name of the target object, and asked “How consistent is the size of the target object compared to the scene?”. First, they were required to select a rating from a 6-point scale; the scale options were: extremely consistent, consistent, slightly consistent, slightly inconsistent, inconsistent, extremely inconsistent. Then, they were asked to give a size direction judgment to further qualify their rating, which was one of either Perfectly sized, Too small, or Too large. The Extremely Consistent rating and Perfectly Sized judgment could only be selected together; if one was selected without the other then the subject was prompted to check the rating and/or size direction judgment before they could move on to the next trial. The six rating options were coded from 0 (extremely consistent) to 5 (extremely inconsistent), and the average rating across subjects was calculated and rounded to the nearest whole number, such that each image was assigned to 1 of 5 groups. Prior to beginning the task, subjects were shown four demo images as examples of the type of images and object scales that they would be presented with; these were images that were not included in the actual experiment but were created in the same style using Unity and GIMP.

### Imaging Procedures

A Siemens PRISMA 3-Tesla (3 T) scanner was used to obtain both the structural and functional scans for each of the participants, using a 64-channel head/neck coil (the neck coils were disabled during the scans, leaving a total of 50 head channels in use). Two detailed structural images were produced for each subject in the first scanning session, which were used to produce a reconstructed 3D brain, to be overlaid with the functional data; T1-weighted (MPRAGE, TE = 2.22 ms, TR = 2500 ms, slice thickness = 0.94 mm, slices = 208, flip angle = 7 degrees, FOV = 241 mm, coverage = full brain, orientation = sagittal (with orientation adjustments where necessary to align down the midline for each subject)) and T2-weighted (TE = 566 ms, TR = 3200 ms, slice thickness = 0.94 mm, slices = 208, FOV = 241 mm, coverage = full brain, orientation = sagittal (with orientation adjustments matched to the T1 parameters). Localizer scans were also performed within this first session. These were used to produce functionally defined regions of interest (ROIs); see the Localizer Scans section below for details of stimuli. The second session contained only the main experiment scans; five repeated scans, each lasting approximately 10 min, were performed within the session, such that the total session time was less than 1 hour. Short breaks within the scanner were encouraged between each scan to avoid subject fatigue. For all the functional scans (localizers and the main experiment), multi-band imaging was used to allow shorter TR lengths of 700 ms, for 2 mm^3^ isotropic voxels, which covered the full brain (EPI scans, TE = 36 ms, TR = 700 ms, slice thickness = 2 mm, slices = 72, FOV = 208 mm, flip angle = 52 degrees, Multi-band acceleration factor = 8, coverage = full brain, slice orientation = transversal (with orientation adjustments where necessary to align down the midline for each subject)). The protocols for the multi-band scanning were obtained by UCSB’s Brain Imaging Centre (BIC) from The Center for Magnetic Resonance Research (CMRR), University of Minnesota (http://www.cmrr.umn.edu/).

### fMRI experiment design

A rapid event-related design was used. The scene (without the target object) was presented for a randomized period between 2 and 3.5 s, with a fixation bullseye located where the target object should be (0.2° diameter gray circle, overlaid with a black 0.13° diameter circle). This was implemented to allow some adaptation to the scene prior to the onset of the target object (i.e., to maximize the response to the target object itself and not just the properties contained within the scene). The images were always positioned so that the target object location was in the center of the screen, to minimize eye movements. After the randomized period, the fixation point was replaced by the target object on the scene for 500 ms, followed by a 500 ms blank period (with fixation point) prior to the next scene onset (see Fig. 1e for an example of a single trial). To help the subjects maintain attention throughout the scans, we used an attentional task. Subjects were asked to press a button if the fixation point was *not* replaced by an object, i.e., the fixation point would just disappear from the scene without being replaced by anything else. There were 20 of these task trials presented throughout a scan, which were created using the fixed FOV scenes from each of the ten target object scenes, each presented twice throughout the scan. The 120 test images (10 target objects x 12 conditions) and 20 task images were presented across ten blocks (14 trials per block), with a 10 s blank period (containing a fixation point) between each block. Each block contained 2 task trials, which were presented at random points within the block. The object’s retinal size and scene field-of-view manipulations were presented in different blocks, which were alternated, i.e., a block containing scene field-of-view manipulated images, followed by a block of object retinal size manipulation images, etc. The image presentation order was randomized for each condition manipulation set prior to splitting into blocks – the only restriction implemented was that the same target object could not be presented for consecutive trials. Five scans were performed for each subject.

### Localizer scans

Functional regions of interest were defined using the output of two localizer scans. To identify the intraparietal sulcus (IPS) visual area, which responds to (amongst other things) eye movements, we had subjects carry out a single 5-minute scan, the IPS localizer. This contained alternating blocks of stationary and moving fixation points (8 blocks per condition). For the blocks of stationary fixation points, the fixation point stayed in the center of the screen for 20 s, and for the blocks of moving fixation points, the point moved from left to right on the screen every 500 ms (to randomized positions along the horizontal axis) for 20 s. Subjects were instructed to fixate on the point throughout the scan (i.e., both when stationary and when moving).

Face, scene and object regions were identified using contrasts produced from a single localizer scan, referred to as the LFP localizer because it contains all the stimuli necessary for identifying regions LO, FFA, and PPA (amongst other similarly-defined areas)^27^. This scan presented five 18-second blocks of four conditions: faces, scenes, objects, and scrambled objects. Each block contained 18 images, which were different in each block of the same condition (image presentation 300 ms, with 700 ms ISI). Images were 14° × 14° visual angle and were located centrally on the screen with a 0.2 diameter black filled fixation point overlaid in the center, which the subjects were required to fixate. To maintain attention throughout the scan, we had subjects perform a simple one-back task. They were instructed to press a button if they were presented with the same image twice in a row. Three 12-second blank fixation periods were distributed across the start, middle, and end of the scan.

### fMRI data processing

A model of each subject’s brain was reconstructed from the structural scans, using a combination of FSL (http://fsl.fmrib.ox.ac.uk/fsl/fslwiki/)^68^ and Freesurfer (http://surfer.nmr.mgh.harvard.edu/)^69,70^, using the recon-all function. ITKGray (http://web.stanford.edu/group/vista/cgi-bin/wiki/index.php/Software#ITKGray adapted and developed from ITKSnap^71^ by R.F. Dougherty, Stanford University) was used to check and resolve the reconstructed brains for any handles and cavities. The 2017 version of the VISTA software (https://web.stanford.edu/group/vista/cgi-bin/wiki/index.php/Software) (Vista Lab, Stanford University), running on MATLAB R2016b (The MathWorks Inc., Natick, MA, USA), was used for processing the functional data. Gray and white matter were segmented during the reconstruction process so that analyses could be restricted to the segmented cortical gray matter within the VISTA software^72^.

The functional scans were processed using mrVista (from the VISTA software), running on MATLAB R2016b. This software was also used to perform motion correction between and within functional scans from each session using a maximum likelihood alignment routine^73^. An average of the first functional scan within each session was used to align the functional data to the structural reconstruction of the brain.

### Functionally defined ROIs

**Fig. 6:**
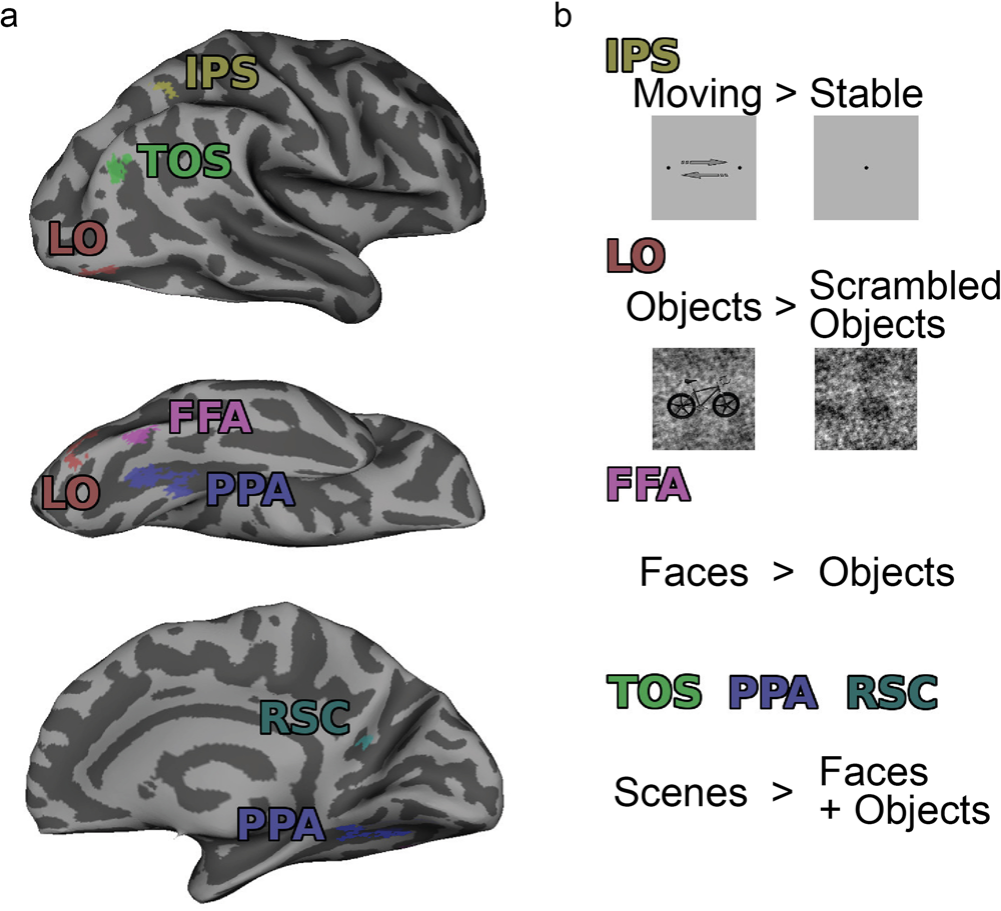
Functional ROI examples. **a** Example of ROI locations in one subject, shown on an inflated mesh of the right hemisphere. **b** Contrasts from the localizer scans used to identify each ROI are shown, along with some examples of the stimuli. IPS was identified with the IPS localizer, and all other ROIs were identified from the LFP localizer; see Methods for details.

The majority of ROIs used in this study were functionally defined on an individual subject level: TOS, PPA, RSC, IPS, LO, and FFA. First, General Linear Model (GLM) analyses were performed on the data from the IPS and LFP localizers; then contrast maps were produced to identify areas with the greatest functional activity to specific conditions. Active voxels were restricted to those that were significant to *p* < 10^−8^. The contrasts used to identify each region were as follows: intraparietal sulcus (IPS), moving > stationary; fusiform face area (FFA), faces > objects; parahippocampal place area (PPA), retrosplenial cortex (RSC), and transverse occipital sulcus (TOS), scenes > faces + objects; lateral occipital visual area (LO), objects > scrambled objects. An example of the ROI locations and the contrasts used to define each of the ROIs are shown in Fig. 6.

The location of each ROI was initially determined by identifying the approximate anatomical locations from slices of the sagittal, coronal and axial planes (the Gray View in mrVista), based on locations identified in previous research^23,27,74–78^. Flat maps of the gray matter were then created around each of these likely ROI areas using mrVista, to visualize a flattened 2D view of the contrast maps. From the flat maps, and for each ROI, an outline was drawn around the active voxel clusters (thresholded to *p* < 10^−8^ on the relevant contrast maps) that best aligned with the likely anatomical location of each ROI, this outline ROI was then back-projected from the flat view into the functional data space of the gray view, and restricted such that the ROI clung to the edges of the active voxels within the segmented gray matter.

For every functionally defined ROI, top activation voxels were defined for use in the MVPA analysis. This categorization was performed using mean z scores for all the scene-onset events (i.e., the start of the scene presentation for each trial, rather than the actual onset of the object within the scene). Baseline z scores were calculated within each voxel (*z*_*n*_) from the linear detrended time-series for each scan (*d*_*n*_), using the mean (*b*) and standard deviation (*s*_*b*_) of the blank periods within each scan (*z*_*n*_ = (*d*_*n*_−*b*)/*s*_*b*_). Z scores for each scene-onset event were extracted using a fixed HRF peak delay of ~5 s (7TRs = 4.9 s). The mean z scores across events and scans for each voxel were calculated, and the 50 voxels within each ROI that had the highest mean z scores were considered the most responsive voxels/top activation voxels (all voxels were kept if there were fewer than 50 voxels total for a given ROI).

### Anatomically defined ROI

In order to have an ROI that represented early visual areas, and in the absence of retinotopic scans for our participants, we created an anatomically defined V1 ROI for each individual. This was done with the Benson Atlas^79^ using the Neuropythy Python Library run in Python 2.7.12, which was applied to the reconstructed brain images acquired from Freesurfer with the *recon-all* function. The V1 atlas produced by the Benson Atlas covers the entirety of the visual field, and therefore to better represent the visual space that our visual stimuli occupied, we restricted this ROI using a number of wedges spanning different eccentricities in each hemisphere. Our rectangular stimulus images were 10 degrees × 5.84 degrees, but since the fixation point of each image was always centered on the object location, rather than on the center of the scene, the entire scene varied slightly in its position on the retina between trials; because of this, it would not be possible to produce an ROI that never covered any of the gray background whilst also containing all of the target objects. Therefore, we created an anatomical V1 ROI that combined several wedges of different eccentricity (in each hemisphere) to approximately fill the rectangular stimulus area. Restricting the anatomical V1 ROI in this way serves to ensure that the entirety of all objects is always within the ROI, despite often also including some parts of the gray background.

The final ROI for each subject was combined across hemispheres and comprised five wedges for each hemisphere: the first extended from 0 to 180 degrees polar angle and to 2.9 degrees visual angle eccentricity (i.e., to the approximate height (5.84 degrees) of the stimulus, from the center of the image), the last wedge extended from 55 to 125 degrees polar angle and to 5 degrees eccentricity (i.e., to the width (10 degrees) of the stimulus, from the center of the image). The three additional wedges were evenly spaced between the first and last wedges (at 3.425, 3.95, and 4.475 degrees eccentricities, with polar angles of 32–148, 43–137, and 50–130 degrees polar angle, respectively). These wedges were created and combined using the output of the Benson Atlas, with the Freesurfer commands mri_vol2label, mri_binarize, mris_label_calc, mri_label2vol, and mri_convert, as well as fslmaths from FSL.

As with the functionally-defined ROIs, top activation voxels were defined for the V1 ROI for use in the MVPA analysis. These were defined in the exact same way as for the functional ROIs, by calculating the mean z scores across all scene-onset events and scans for each voxel, and then taking the top 50 voxels within the ROI that had the highest mean z scores (all voxels were kept if there were fewer than 50 voxels).

### Statistics and reproducibility

#### GLM analysis

We used a General Linear Model (GLM) with Rank-1 constraint with a Finite Impulse Response (FIR) basis function (R1-GLM with FIR), as described and implemented in the Python package *hrf_estimation* by Pedregosa^30^ (https://github.com/fabianp/hrf_estimation). This method produces an HRF estimate that is equal across conditions, i.e., one HRF estimate is produced for each voxel, with activation coefficients produced for each condition, within each voxel; the name stems from the constraint applied to the vector of coefficients, whereby they must lie within the space of rank one matrices^30,80^. Compared to other GLM methods of accounting for individual voxel HRF estimates, Pedregosa et al.^30^ demonstrate that with FIR (along with an R1-GLM using separate designs (R1-GLMS) and with FIR basis), the encoding and decoding accuracy was greater than other traditional GLM methods. The activation coefficients outputted for each model—the beta weights, as reported here—were averaged across voxels for each condition, within each ROI, for each subject, prior to producing group averages across subjects for each ROI.

This analysis was performed twice—once using the six scale consistency levels as the conditions and once with the levels split further, by the direction of mis-scaling (mis-scaled to be too large vs. too small). For each, we measured the difference between the mean beta weight for the normal scale consistency level(s) and the mean of the scale inconsistent levels (i.e., the mean across the remaining five scale consistency levels). We tested whether the normal scale consistency level was significantly greater than the scale-inconsistent (mis-scaled) levels using permutation tests; the scale consistency levels were randomly permuted across events prior to running the GLM analysis, and then the exact same process of averaging across voxels for each ROI and subject was performed before extracting the difference value between the mean beta weights (the permutation difference value). This permutation process was repeated 1000 times, and the *p*-value of our data was determined by the proportion of permutation difference values that exceeded our actual difference value for a given ROI (e.g., <5% would be considered statistically significant). Across ROIs (and scaling directions, for the second analysis), the significance values were false-discovery rate (FDR) corrected (all FDR corrections in this paper were performed using the *fdr_bh* function^81^ in MATLAB, which uses the Benjamini & Hochberg^82^ procedure of FDR correction).

For the anatomically defined V1 ROI, the above Python analyses were run using Python 3.7 on a Debian 10.6 Buster operating system. For all other ROIs these analyses were run using Python 2.7 on Ubuntu 16.04.2 operating system.

#### MVPA

Multi-Voxel Pattern Analysis (MVPA) was performed using a 2-class classifier to predict whether given test events were classed as containing a normally-scaled object or a mis-scaled object. This analysis was performed five times, such that the classes used for training and testing were the normal scale level as well as one of the five mis-scaled levels, in turn; the final percent correct (PC) value for each subject in an ROI was taken as the average PC across all five analysis pairings.

For every subject, baseline z-scores were calculated for each time point in each voxel (*z*_*n*_) from the linear detrended time-series (*d*_*n*_), using the mean (*b*) and standard deviations (*s*_*b*_) of the blank periods of each scan from each voxel:

$${z}_{n}=\frac{({d}_{n}-\underline{b})}{{s}_{b}}$$

The baseline z-score values were then extracted for each stimulus event in each scan (for each voxel), following an event-onset adjustment to account for the peak-delay of the hemodynamic response. This adjustment was performed for each voxel based on the peak delay (the time in TRs to the peak of the HRF) of voxel-specific HRF estimates; these were acquired from an additional R1-GLM with FIR basis function, in which all events were combined into one condition, and all scans were concatenated, before running the model to produce an HRF estimate for each voxel (using the Python package *hrf_estimation* by Pedregosa^30^ (https://github.com/fabianp/hrf_estimation), used for the GLM analysis). These voxel-specific HRFs were also utilized in the voxel-wise encoding model.

A Leave-One-Run-Out Cross-Validation (LORO-CV) method was used, where the classifier was trained on events from 4 out of the 5 scans, and then tested on the 5^th^ scan. This was repeated until each scan had been used as the testing scan. The classifier was trained to make a binary prediction between normal scale and mis-scaled, this used two classes: Class 1 = normal scale condition trials, and Class 0 = trials for one of the five mis-scaled condition levels (the number of trials in each class was balanced). For testing, trials from the same conditions for the left-out scan run (e.g., normal scale and mis-scaled level 1 conditions) were assigned to the two classes. A linear support vector machine classifier was trained and tested (using MATLAB functions *fitcsvm*, with a linear kernel; and *predict*). The testing phase used the output from *fitcsvm* and the event data from the test scan to produce class predictions for each event. The percentage of correct predictions was calculated for each LORO-CV run, and then the mean percent correct (PC) was calculated across all runs for that subject in each ROI (this was done for each of the five analysis pairings, before taking the average across those pairings to get the final PC value for each subject and ROI).

The average PC across subjects was calculated for each ROI, and to determine whether these mean PC values differed significantly from chance, permutation tests were performed. For this, the same process was repeated 1000 times for each subject/ROI; for each permutation, the labeling of the training events was randomized, so that each event was randomly allocated to one of the two classes during the classification stage. The rest of the procedure, for each permutation, was identical to that described above. From the resulting 1000 mean PC values acquired across subjects for each ROI, the significance level was extracted as the proportion of permutation values that were greater than the mean PC values from the data. Significance values were then FDR corrected across ROIs.

#### Voxel-wise encoding

To estimate the contribution of each object property to the fMRI signal, we applied a voxel-wise encoding model to every voxel for every subject. This process applies a regularized linear regression to the events extracted from the linear-detrended time series of every voxel, using a predictor matrix that contains the property level for every object property at each event; property levels were first normalized around the mean, and then between −1 and 1, within each property (see Fig. 4a for schematic illustration). The regression was performed using the *fitrlinear* function in MATLAB. The BOLD signals from the time series of each voxel were extracted for each stimulus event using the peak delay from the voxel-specific HRF estimates (acquired from the same R1-GLM with FIR used in the MVPA analysis, in which all events were combined into one condition, and all scans were concatenated, before running the model to produce an HRF estimate for each voxel (using the Python package *hrf_estimation* by Pedregosa^30^ (https://github.com/fabianp/hrf_estimation))), i.e., the extracted TRs corresponded to the real-time event onset plus the peak-delay in TRs. The predictor matrices and event signals for each of the five scans were concatenated prior to performing the regression for each voxel. This analysis produces a feature weight for each of the inputted object properties for each voxel, which indicates the level of contribution that each property has to the fMRI signal. Permutation tests were used to determine whether the mean feature weights for each property were significantly different to chance: the same process was repeated 1000 times, with the feature levels randomly assigned to events at the start of the encoding model process for each permutation (every voxel in any given permutation used the same randomized feature matrix). The proportion of mean feature weights from the permutations that were larger (or smaller) than the actual mean feature weight determined the *p*-value; since feature weights could either be negative or positive, a two-tailed test was used, so *p* values were corrected by multiplying by two, e.g., if 25 of the 1000 permutations had a larger (or smaller) mean feature weight than the actual data, then *p* = .05 (.025*2). *P* values were then FDR corrected across all properties and ROIs.

For each ROI, we wanted to assess whether the voxels that were most responsive to scale consistency (i.e., those with the highest absolute feature weights for that property), were also particularly *selective* for this property by having a ratio of feature weights that was significantly larger than chance for scale consistency. First, within each ROI, we sorted the voxels by their absolute feature weight values for scale consistency, and then took the top 30% of voxels, i.e., those with the highest feature values (most responsive) for scale consistency relative to other voxels in that ROI (see Supplementary Note 1 for information on different sample sizes used for the top %, and Supplementary Note 2 and Supplementary Figure 2 for information on measuring the selectivity to other features). For each of these voxels, the ratio of absolute feature weights was calculated across properties before averaging across voxels/subjects within each ROI. The mean feature weight ratio for scale consistency in each ROI was then tested for significance against chance using the output of the permutation tests; for each permutation, the same process for extracting the top 30% of voxels and the feature weight ratio was carried out. The proportion of mean feature weight ratio values from the permutations that were larger (or smaller) than the actual mean feature weight ratio for each ROI, determined the *p*-value (as this was also a two-tailed test, a correction was applied to the *p*-value by multiplying by 2). *P* values were then FDR corrected across all properties and ROIs. Figure 4c shows the histograms of the permutation data, with arrows indicating the value of the actual mean feature weight ratio for scale consistency (with significance markers).

To validate the encoding model, we used a LORO-CV method to predict the BOLD signal responses to events in each scan. The encoding model was carried out five times, with one scan (the test scan) being left out in turn; for every voxel, the feature weights produced using the remaining four scans were multiplied by the normalized feature matrix for the test scan (i.e., the property levels for all images, normalized between −1 and 1) to produce a predicted signal. The average signal for each voxel across each run was then calculated, before calculating the average signal for each scale-consistency level (split by direction of mis-scaling, in the manner plotted for the GLM data of Fig. 2b); a schematic of this method is illustrated in Fig. 5a. The mean predicted signal across subjects and ROIs for each condition were normalized, and the difference between the normal scale level and mean of the mis-scaled levels was tested for significance within each ROI for each direction of mis-scaling using paired t-tests (one-tailed, to test whether the normal scale level was significantly greater than the mis-scaled levels) (SPSS Statistics v25, IBM), corrected for multiple comparisons using FDR correction across all 14 tests (two for each of the seven ROIs) (data plotted in Fig. 5b).

### Reporting summary

Further information on research design is available in the Nature Research Reporting Summary linked to this article.

## Supplementary information

Supplementary Material

Description of Additional Supplementary Files

Supplementary Data 1

Supplementary Data 2

Supplementary Data 3

Supplementary Data 4

Reporting Summary

## Data Availability

Source data underlying Figs. 2–5 are presented in Supplementary Data 1-4. All other data generated and analyzed during the current study are available from the corresponding author on reasonable request.

## Code availability

The code used to analyze the data in the current study is available from the corresponding author on reasonable request.
