# Supplementary material for: The transverse occipital sulcus and intraparietal sulcus show neural selectivity to object-scene size relationships
Source: Commun Biol. 2021 Jun 22;4:768. doi: 10.1038/s42003-021-02294-9 (PMC8219818; doi:10.1038/s42003-021-02294-9)
Supplement: Supplementary file 1 — Supplementary Material [file 42003_2021_2294_MOESM1_ESM.pdf]

Supplementary Material

The transverse occipital sulcus and intraparietal sulcus show neural selectivity to object-scene size relationships

Welbourne, Jonnalagadda, Giesbrecht, and Eckstein

Supplementary Table 1 Spearman rank correlations between the scale consistency levels and the other object properties assigned to each stimulus image; with the addition of the correlation between scale consistency and perceived *scale consistency. The values for each object property were grouped into 4 or 5 levels before the Spearman rank correlations were performed - these levels were the same as those used in the voxel-wise encoding model. See Supplementary Figure 1 for a visualisation of the un-grouped property values. Significance values have not been corrected for multiple comparisons, to avoid false negatives in the correlations with scale consistency.*

	Scale Consistency	Object Retinal Size	Real-World Object Size	Scene Field-of-View
Perceived Scale Consistency	$r_s(118) = 0.8799$<br>$\boldsymbol{p = 10^{-39}}$	-	-	-
Object Retinal Size	$r_s(118) = -0.0227$<br>$p = 0.8060$	-	-	-
Real-world object size	$r_s(118) = 0$<br>$p = 1$	$r_s(118) = 0.6240$<br>$\boldsymbol{p = 10^{-13}}$	-	-
Scene Field-of-View	$r_s(118) = -0.0840$<br>$p = 0.3616$	$r_s(118) = -0.4074$<br>$\boldsymbol{p = 10^{-5}}$	$r_s(118) = -0.2312$<br>$\boldsymbol{p = 0.0111}$	-

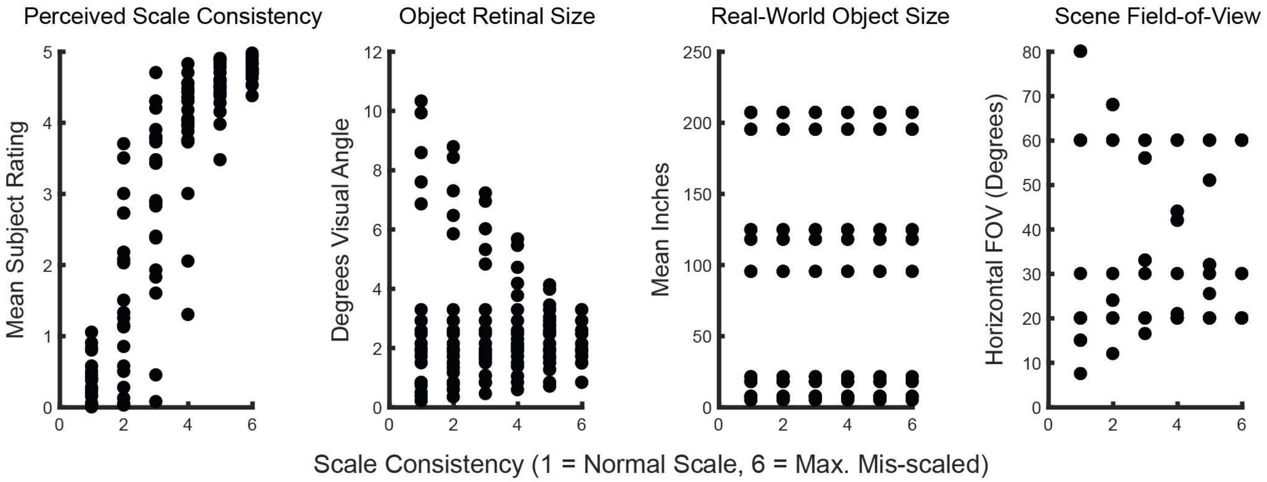

Supplementary Figure 1 Scatter plots of the different object property values for each image, plotted against scale consistency, which were used to create each image. Note that for the real-world object size and the scene field-of-view properties, multiple images had the same property value, and as such it appears that there are fewer data points plotted (when actually there are several overlaid). Unlike the other properties, Perceived Scale Consistency was not used as one of the additional object properties in the voxel-wise encoding model analysis; these values were acquired to indicate how well our scale consistency levels correlated with user ratings of perceived scale consistency (see Supplementary Table 1 for correlation).

Supplementary Note 1: Sample Sizes for the 'most responsive' voxels in the Voxel-wise encoding model

For each ROI, we wanted to assess whether the voxels that were most responsive to scale consistency (i.e. those with the highest absolute feature weights for that property), were also particularly *selective* for this property, by having a ratio of feature weights that was significantly larger than chance for scale consistency. To do this, within each ROI we sorted the voxels by their absolute feature weight values for scale consistency, and then took the top 30% of voxels to use in a feature weight ratio analysis (see Results and Methods), i.e. those with the highest feature values (most responsive) for scale consistency relative to other voxels in that ROI. We repeated this analysis for different sample sizes of top voxels; we conducted the same process in steps of 5%, from the top 5% through to the top 50%. Between the top 25% and 50% of voxels, we found the same ROIs showed significant selectivity as were reported in the Results (TOS, IPS, and V1; all at the same significance levels as reported in the Results, with the exception of

TOS, which increased significance to $p=.007$ for the top 40% and $p<.001$ for the top 45% and 50%). For levels 45% and 50% PPA selectivity to scale consistency was significantly greater than chance, at $p=.035$ and $p=.025$, respectively. For the top 5%, LO selectivity to scale consistency was significantly lower than chance, at $p=.035$. The selectivity in V1 was significant at $p<.001$ for all of the levels tested (in each case, significantly lower selectivity than chance). For all reported significance levels, there were 1000 permutations, a two-tailed test, and FDR correction across ROIs.

Supplementary Note 2: Selectivity of ROIs for other features

The process of measuring the selectivity of ROIs was also carried out for the other features: retinal size, real-world size, and scene field-of-view. The same process described in the Methods for measuring selectivity to scale consistency was carried out for each of the other features, i.e. using the top 30% of voxels most responsive to each feature (*Supplementary Figure 2*). No significant selectivity was found in any of the ROIs for retinal size. PPA ($p<.001$), TOS ($p=.011$) and V1 ($p<.001$) all showed significant selectivity to real-world size, and LO was significantly *not* selective to real-world size ($p=.005$) (mean feature weight ratio significantly lower than expected by chance). LO ($p=.007$) and V1 ($p<.001$) both showed significant

selectivity to scene field-of-view. For each feature, significance was determined using the permutations (1000 permutations, two-tailed, FDR corrected across ROIs).

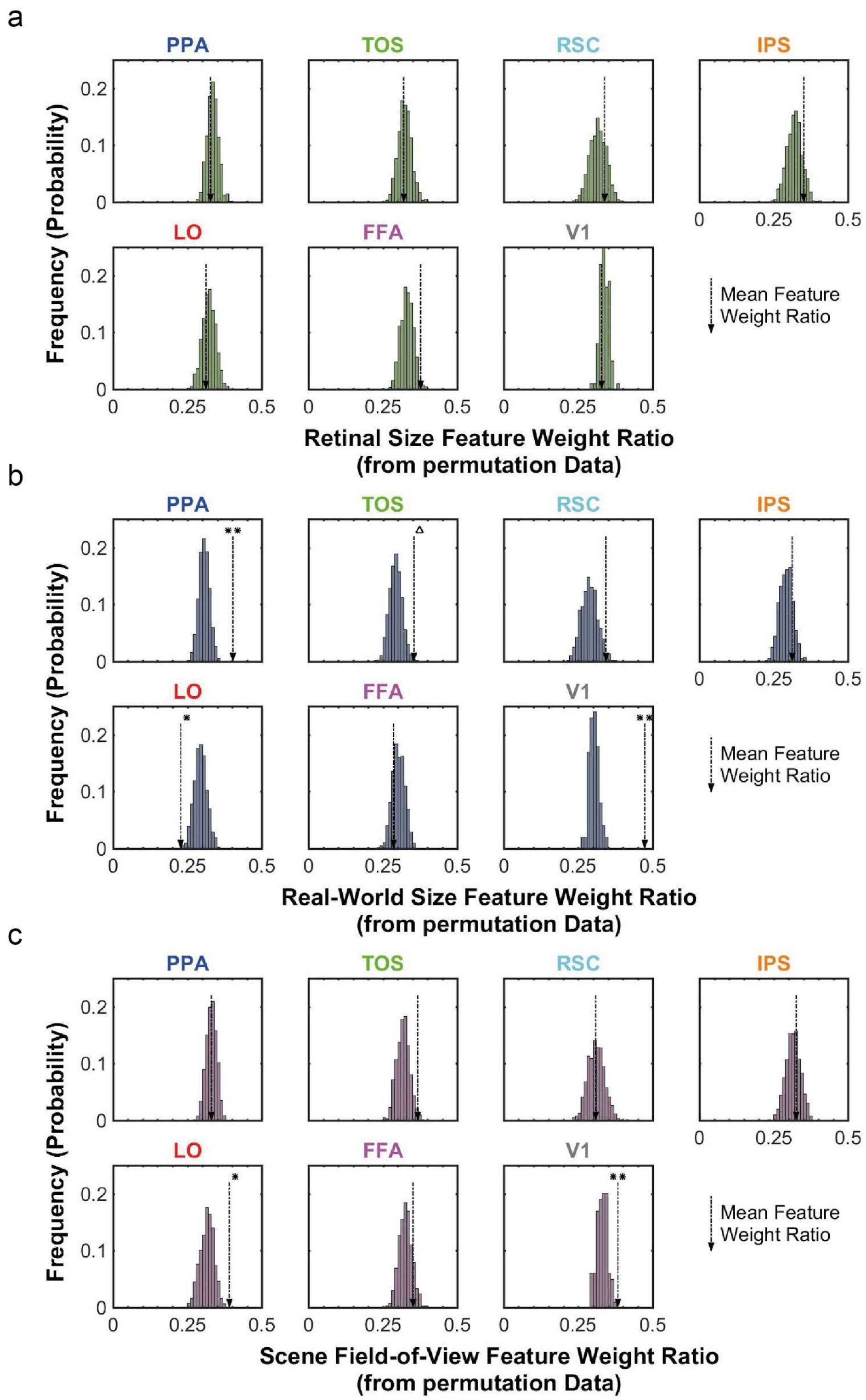

*Supplementary Figure 2 Selectivity of each feature: Histograms of the permutation data (n=1000) for each feature weight ratio, a) retinal size, b) real-world size, c) scene field-of-view. For each feature the top 30% of voxels most responsive to that feature was used. The mean feature weight ratio for the corresponding feature from the actual data is indicated by dashed arrows, with significance indicated where relevant: **p<.001, *p<.01, △p<.05, 1000 permutations, two-tailed, FDR corrected across ROIs.*
