# Supplementary material for: The transverse occipital sulcus and intraparietal sulcus show neural selectivity to object-scene size relationships
Source: Commun Biol. 2021 Jun 22;4:768. doi: 10.1038/s42003-021-02294-9 (PMC8219818; doi:10.1038/s42003-021-02294-9)
Supplement: Supplementary file 2 — Description of Additional Supplementary Files [file 42003_2021_2294_MOESM2_ESM.pdf]

Description of Additional Supplementary Files

File Name: Supplementary Data 1
Description: Data for Figure 2

File Name: Supplementary Data 2
Description: Data for Figure 3

File Name: Supplementary Data 3
Description: Data for Figure 4

File Name: Supplementary Data 4
Description: Data for Figure 5
