# Supplementary material for: The transverse occipital sulcus and intraparietal sulcus show neural selectivity to object-scene size relationships
Source: Commun Biol. 2021 Jun 22;4:768. doi: 10.1038/s42003-021-02294-9 (PMC8219818; doi:10.1038/s42003-021-02294-9)
Supplement: Supplementary file 7 — Reporting Summary [file 42003_2021_2294_MOESM7_ESM.pdf]

nature research

Corresponding author(s): Miguel Eckstein

Last updated by author(s): 26/04/20

Reporting Summary

Nature Research wishes to improve the reproducibility of the work that we publish. This form provides structure for consistency and transparency in reporting. For further information on Nature Research policies, see our Editorial Policies and the Editorial Policy Checklist.

Statistics

For all statistical analyses, confirm that the following items are present in the figure legend, table legend, main text, or Methods section.

n/a	Confirmed	
☐	☒	The exact sample size (*n*) for each experimental group/condition, given as a discrete number and unit of measurement
☐	☒	A statement on whether measurements were taken from distinct samples or whether the same sample was measured repeatedly
☐	☒	The statistical test(s) used AND whether they are one- or two-sided<br>*Only common tests should be described solely by name; describe more complex techniques in the Methods section.*
☐	☒	A description of all covariates tested
☐	☒	A description of any assumptions or corrections, such as tests of normality and adjustment for multiple comparisons
☐	☒	A full description of the statistical parameters including central tendency (e.g. means) or other basic estimates (e.g. regression coefficient) AND variation (e.g. standard deviation) or associated estimates of uncertainty (e.g. confidence intervals)
☐	☒	For null hypothesis testing, the test statistic (e.g. *F*, *t*, *r*) with confidence intervals, effect sizes, degrees of freedom and *P* value noted<br>*Give P values as exact values whenever suitable.*
☒	☐	For Bayesian analysis, information on the choice of priors and Markov chain Monte Carlo settings
☒	☐	For hierarchical and complex designs, identification of the appropriate level for tests and full reporting of outcomes
☒	☐	Estimates of effect sizes (e.g. Cohen's *d*, Pearson's *r*), indicating how they were calculated

Our web collection on statistics for biologists contains articles on many of the points above.

Software and code

Policy information about availability of computer code

Data collection	Stimuli used in these experiments were computer-generated using Unity (Unity Technologies) and GIMP v2.8 (https://www.gimp.org/). Amazon Mechanical Turk and Qualtrics (Qualtrics, Provo, Utah, USA) was used to collect behavioural data from a separate sample of 240 subjects (that were not used in the fMRI experiment). A Siemens 3-Tesla scanner was used to collect the MRI and fMRI data - protocols for multi-band scanning were obtained by UCSB's Brain Imaging Centre from The Centre for Magnetic Resonance Research (CMRR), University of Minnesota (http://www.cmrr.umn.edu/). Stimuli were presented in the scanner using MATLAB R2016b (The MathWorks Inc, Natick, MA, USA) and the Psychophysics Toolbox (Brainard, 1997, https://doi.org/10.1163/156856897X00357).
Data analysis	A model of each subject's brain was reconstructed from the structural scans, using a combination of FSL (http://fsl.fmrib.ox.ac.uk/fsl/fslwiki/), Freesurfer (http://surfer.nmr.mgh.harvard.edu/), ITKGray (http://web.stanford.edu/group/vista/cgi-bin/wiki/index.php/Software#ITKGray adapted and developed from ITKSnap by R.F. Dougherty, Stanford University), and the 2017 version of VISTA software (https://web.stanford.edu/group/vista/cgi-bin/wiki/index.php/Software) (Vista Lab, Stanford University), running on MATLAB R2016b (The MathWorks Inc., Natick, MA, USA). The functional scans were processed using mrVista (from the VISTA software), running on MATLAB R2016b.<br><br>An anatomically-defined area (V1) was acquired using the Benson Atlas (Benson, N. C. et al. The Human Connectome Project 7 Tesla retinotopy dataset: Description and population receptive field analysis. J. Vis. 18, 23–24 (2018)) using the Neuropythy Python Library run in Python 2.7.12, which was applied to the reconstructed brain images acquired from Freesurfer. The final V1 region of interest used was created and combined using the output of the Benson Atlas, with the Freesurfer commands mri_vol2label, mri_binarize, mris_label_calc, mri_label2vol, and mri_convert, as well as fslmaths from FSL.<br><br>The General Linear Model (R1-GLM with FIR) was implemented using the Python Package 'hrf_estimation' by Pedregosa (2015) (https://github.com/fabianp/hrf_estimation). The Multi-Voxel Pattern Analysis (MVPA) was performed using the MATLAB functions 'fitcsvm' (with a linear kernel) and 'predict'. The regression analysis used in the voxel-wise encoding model was performed using the 'fitrlinear' function in MATLAB.

For the anatomically-defined V1 ROI, the above Python analyses were run using Python 3.7 on a Debian 10.6 Buster operating system. For all other ROIs these analyses were run using Python 2.7 on Ubuntu 16.04.2 operating system.

All independent and paired T-tests were performed using SPSS Statistics v25 (IBM). All false discovery rate (FDR) corrections were performed within MATLAB using the 'fdr_bh' function (https://uk.mathworks.com/matlabcentral/fileexchange/27418-fdr_bh). All other statistical processing, such as averaging, calculating standard errors, and running permutation tests, was performed within MATLAB.

For manuscripts utilizing custom algorithms or software that are central to the research but not yet described in published literature, software must be made available to editors and reviewers. We strongly encourage code deposition in a community repository (e.g. GitHub). See the Nature Research guidelines for submitting code & software for further information.

Data

Policy information about availability of data

All manuscripts must include a data availability statement. This statement should provide the following information, where applicable:

- Accession codes, unique identifiers, or web links for publicly available datasets
- A list of figures that have associated raw data
- A description of any restrictions on data availability

The datasets generated and analysed during the current study are available from the corresponding author on reasonable request.

Field-specific reporting

Please select the one below that is the best fit for your research. If you are not sure, read the appropriate sections before making your selection.

☐ Life sciences ☒ Behavioural & social sciences ☐ Ecological, evolutionary & environmental sciences

For a reference copy of the document with all sections, see nature.com/documents/nr-reporting-summary-flat.pdf

Behavioural & social sciences study design

All studies must disclose on these points even when the disclosure is negative.

Study description	This study was a quantitative experiment.
Research sample	Volunteers were either from the University of California, Santa Barbara or found out about the study via members of the research group. Of the 14 subjects used in the data analysis, the mean age was 22.8 years (range 19 -28), and there were 8 female and 6 male subjects. The number of subjects used is representative of samples used in similar research designs.
Sampling strategy	The number of subjects used is representative of samples used in similar research designs. No statistical methods were used to predetermine the sample size used.
Data collection	In the MRI experiment, a Siemens 3T scanner was used, and stimuli were presented via a projector and mirror to the subject (stimuli presented on a computer using MATLAB). During the scans the researcher and MRI operator were present in the control room of the scanner. For behavioural data collected via Amazon Mechanical Turk, the stimuli were presented using Qualtrics (Qualtrics, Provo, Utah, USA) on the subjects' own home computers; subjects provided informed consent online prior to completing the task - no researchers were present with these subjects during the task completion, but researchers were available to be contacted with any questions and the subjects were provided with experiment information. Prior to the tasks the subjects were blind to the hypotheses of the experiment, but were given detailed instructions on their task and shown example images before taking part. Participants were debriefed about the experiment hypotheses after the tasks.
Timing	March - May 2017
Data exclusions	1 participant was excluded from the analysis (15 participants recruited, 14 used) due to large motion during fMRI scans - the motion criteria was predetermined prior to analysing the data. In addition to movement this subject reported falling asleep during multiple scans.
Non-participation	0 participants dropped out of the study.
Randomization	All subjects took part in all experimental conditions - a within-subject design was used.

Reporting for specific materials, systems and methods

We require information from authors about some types of materials, experimental systems and methods used in many studies. Here, indicate whether each material, system or method listed is relevant to your study. If you are not sure if a list item applies to your research, read the appropriate section before selecting a response.

Materials & experimental systems

n/a	Involved in the study
☒	☐ Antibodies
☒	☐ Eukaryotic cell lines
☒	☐ Palaeontology and archaeology
☒	☐ Animals and other organisms
☐	☒ Human research participants
☒	☐ Clinical data
☒	☐ Dual use research of concern

Methods

n/a	Involved in the study
☒	☐ ChIP-seq
☒	☐ Flow cytometry
☐	☒ MRI-based neuroimaging

Human research participants

Policy information about studies involving human research participants

Population characteristics	See above.
Recruitment	The experiment was advertised to undergraduates and postgraduates within UCSB, as well as by word-of-mouth to associates. Individuals either received payment or course credit for their time. Individuals were not given specific information about the hypotheses of the study beforehand (and as such, none of the authors were used as participants in this study), in order to reduce a bias of anticipated responses.
Ethics oversight	The study was approved by the Human Subjects Committee at the University of California, Santa Barbara.

Note that full information on the approval of the study protocol must also be provided in the manuscript.

Magnetic resonance imaging

Experimental design

Design type	The localiser scans used in this study (to identify regions of interest) used a block design. Our main experiment used a rapid event-related design, where the events were split across 10 blocks and were separated by blank periods.
Design specifications	Main Experiment: a total of 140 trials (120 trials plus 20 attentional-task trials) were split across 10 blocks (14 trials per block). A 10 second blank period was presented between each block. A randomised presentation period was used prior to our event presentation, and so the length of each scan could vary between 8 and 12 minutes (typically lasting around 10minutes). Five scans were performed for each subject. These were performed in a single session, which was separate to the session where the localiser scans and structural scans (described below) were obtained.<br><br>Localiser scans:<br>'LFP' localiser scan: This scan contained four conditions which were split into their own blocks. Five blocks were carried out for each condition, lasting 18 seconds each. Three 12 second blank periods were distributed across the start, middle, and end of the scan. The total scan length was approximately 6.5 minutes.<br>'IPS' localiser scan: This scan contained two conditions, presented in their own blocks. There were eight blocks per condition, each lasting 20 seconds. This scan lasted approximately 5 minutes.<br><br>Structural scans:<br>High resolution T1 and T2 scans were obtained for each subject, each lasting approximately 5 minutes. These scans were obtained in the same session as the localiser scans.
Behavioral performance measures	The attentional task used in the fMRI experiments were designed purely to occupy the subject during the scan and encourage attention to the stimuli - a button press was required when a particular event occurred. The task events occurred on trials that were not used in the analysis, and responses were not analysed as part of this study (though the data is available).

Acquisition

Imaging type(s)	Functional (multi-band protocol) scans and structural (T1 and T2) scans were acquired.
Field strength	3T
Sequence & imaging parameters	A 64-channel head/neck coil was used (the neck coils were disabled during the scans, leaving a total of 50 head channels in use).<br><br>Functional scans: EPI scan, multi-band imaging with an acceleration factor of 8, TR = 700ms, TE = 36.00ms, voxel size = 2x2x2mm, 72 slices, Field of View = 208 mm, Matrix size = 208 x 208 x 144mm, Flip Angle = 52deg, orientation = T>C-18.1, phase encoding direction = A >> P

	Structural Scans:<br>T1 mprage: voxel size = 0.9x0.9x0.9mm, Field of view = 241mm, TR = 2500ms, TE = 2.22ms, slices = 208, flip angle = 7degrees, orientation = sagittal, slice thickness = 0.94mm, phase encoding direction = A >> P<br>T2 space: voxel size = 0.9x0.9x0.9mm, Field of view = 241mm, TR = 3200ms, TE = 566ms, slices =208, orientation = sagittal, slice thickness = 0.94mm, phase encoding direction = A >> P
Area of acquisition	Whole brain scan.

Diffusion MRI ☐ Used ☒ Not used

Preprocessing

Preprocessing software	A model of each subject's brain was reconstructed from the structural scans (T1 and T2), using a combination of FSL (http://fsl.fmrib.ox.ac.uk/fsl/fslwiki/) and Freesurfer (http://surfer.nmr.mgh.harvard.edu/) using the recon-all function. ITKGray (http://web.stanford.edu/group/vista/cgi-bin/wiki/index.php/Software#ITKGray adapted and developed from ITKSnap by R.F. Dougherty, Stanford University) was used to check and resolve the reconstructed brains for any handles and cavities.<br><br>An anatomically-defined area (V1) was acquired using the Benson Atlas (Benson, N. C. et al. The Human Connectome Project 7 Tesla retinotopy dataset: Description and population receptive field analysis. J. Vis. 18, 23–24 (2018)) using the Neuropythy Python Library run in Python 2.7.12, which was applied to the reconstructed brain images acquired from Freesurfer. The final V1 region of interest used was created and combined using the output of the Benson Atlas, with the Freesurfer commands mri_vol2label, mri_binarize, mris_label_calc, mri_label2vol, and mri_convert, as well as fslmaths from FSL.<br><br>The 2017 version of VISTA software (https://web.stanford.edu/group/vista/cgi-bin/wiki/index.php/Software) (Vista Lab, Stanford University) was used, running on MATLAB R2016b (The MathWorks Inc., Natick, MA, USA). The functional scans were processed using mrVista (from the VISTA software) (running on MATLAB R2016b). Alignment of structural scans to a functional inplane scan (a mean of the first functional scan of a session), was performed using the rxAlign feature of mrVista (automatic alignments followed by fine manual point alignment). Motion Correction was performed between and within functional scans using a maximum likelihood alignment routine, built into the mrVista software. No spatial blurring was carried out.
Normalization	Subject data was not normalised to a mean brain - instead, individual functionally-defined regions of interest (ROIs) were identified for each subject using the localiser scan data. ROIs were identified using contrast maps from the localiser scan conditions - active voxels were restricted to those that were significant to p<10^-8, and locations of ROIs were first determined using approximate anatomical locations (guided by previous research, see references in the manuscript), flat maps were then created around these regions in mrVista, and an outline ROI was drawn around the ROI (indicated by active voxel clusters) before being back-projected out of the flat-map and restricted to active voxels only within the segmented gray matter (all tools within standard mrVista functionality). This method produces subject-specific functionally-defined maps of comparable regions within each individual, rather than relying on rough anatomically defined locations from an average brain.
Normalization template	Data were not normalised to a template.
Noise and artifact removal	Motion correction was performed between and within functional scans using a maximum likelihood alignment routine, built into the mrVista software.
Volume censoring	No volumes were censored.

Statistical modeling & inference

Model type and settings	A univariate General Linear Model (R1-GLM with FIR) was implemented using the Python Package 'hrf_estimation' by Pedregosa (2015) (https://github.com/fabianp/hrf_estimation). A multi-variate Multi-Voxel Pattern Analysis (MVPA) was performed using the MATLAB functions 'fitcsvm' (with a linear kernel) and 'predict'. The regression analysis used in the voxel-wise encoding model was performed using the 'fitrlinear' function in MATLAB.
Effect(s) tested	We tested the difference between conditions using permutation tests, to establish whether any differences in mean values were likely to be found by chance. This was done for each ROI, and significance values were all FDR corrected.

Specify type of analysis: ☐ Whole brain ☒ ROI-based ☐ Both

Anatomical location(s)	ROIs were identified using contrast maps from the localiser scan conditions - active voxels were restricted to those that were significant to p<10^-8, and locations of ROIs were first determined using approximate anatomical locations (guided by previous research, see refs 26,30,62-66 in the manuscript), flat maps were then created around these regions in mrVista, and an outline ROI was drawn around the ROI (indicated by active voxel clusters) before being back-projected out of the flat-map and restricted to active voxels only within the segmented gray matter (all tools within standard mrVista functionality). This method produces subject-specific functionally-defined maps of comparable regions within each individual.<br><br>An additional anatomically-defined area (V1) was acquired using the Benson Atlas (Benson, N. C. et al. The Human Connectome Project 7 Tesla retinotopy dataset: Description and population receptive field analysis. J. Vis. 18, 23–24 (2018)) using the Neuropythy Python Library run in Python 2.7.12, which was applied to the reconstructed brain images acquired from Freesurfer. The eccentricity and polar angle covered by the V1 ROI was restricted to most closely match our stimulus, by combining 5 wedges from each hemisphere: the first extended from 0-180 degrees polar angle and to 2.9 degrees visual angle eccentricity, the last wedge extended from 55-125 degrees polar angle and to 5 degrees eccentricity, and the three additional wedges were evenly spaced between the first and last wedges (at 3.425, 3.95, and 4.475 degrees eccentricities, with polar angles of 32-148, 43-137, and 50-130 degrees polar angle, respectively). These wedges were created and combined using the output of the Benson Atlas, with the Freesurfer commands mri_vol2label, mri_binarize, mris_label_calc, mri_label2vol, and mri_convert, as well as fslmaths from FSL.
Statistic type for inference<br>(See Eklund et al. 2016)	Voxel-wise analyses were performed. With averages being taken across voxels within each ROI for each subject.
Correction	False Discovery Rate (FDR) corrections were applied to our significance values, using the 'fdr_bh' function in MATLAB (https://uk.mathworks.com/matlabcentral/fileexchange/27418-fdr_bh).

Models & analysis

n/a	Involved in the study
☒	☐ Functional and/or effective connectivity
☒	☐ Graph analysis
☐	☒ Multivariate modeling or predictive analysis

Multivariate modeling and predictive analysis	A multi-variate Multi-Voxel Pattern Analysis (MVPA) was performed using the MATLAB functions 'fitcsvm' (with a linear kernel) and 'predict'. The model classified trials into one of two classes; training data used 4 of the 5 scans, with each left-out scan used as the test scan in turn (an even number of events were allocated to each class for the training), i.e. a leave-one-run-out cross validation (LORO-CV) method was used.<br><br>The regression analysis used in the voxel-wise encoding model was performed using the 'fitrlinear' function in MATLAB. A feature matrix was produced using four object property values for each event, these features were each normalised around a zero mean and between -1 and 1. As above, an LORO-CV method was used in the validation stages of this model to make predictions about the expected bold signal of each 'left-out' scan.
